# A LisH-domain protein interaction map reveals a Lis1-ARIH2-dynein regulatory axis

**DOI:** 10.1016/j.isci.2025.113912

**Published:** 2025-10-30

**Authors:** Devanshi Gupta, Subbareddy Maddika

**Affiliations:** 1 Laboratory of Cell Death & Cell Survival, Centre for DNA Fingerprinting and Diagnostics (CDFD), Uppal, Hyderabad, Telangana 500039, India; 2 Graduate Studies, Regional Centre for Biotechnology, Faridabad, Haryana 121001, India

**Keywords:** Biochemistry, Proteomics

## Abstract

LisH-domain-containing proteins are involved in diverse cellular processes and disease mechanisms, yet their functional interaction landscape remains poorly characterized. Here, we employed a proteomics-based strategy to systematically map the interaction network of 27 LisH-domain-containing proteins, uncovering 1,410 high-confidence interactions—90% of which are previously unreported. This network reveals unanticipated roles for LisH proteins in cellular regulation and uncovers links to human disease, including cancer-associated interactions. Focusing on Lis1, a cytoplasmic dynein regulator, we identify the RBR-E3 ubiquitin ligase ARIH2 as a key functional interactor. We show that Lis1 promotes ARIH2 deneddylation via the COP9 signalosome (CSN), modulating its ubiquitin ligase activity. Active, neddylated ARIH2 ubiquitinates dynein intermediate chain 1 (DIC1), thus facilitating dynein function in proper organelle positioning. Our findings provide a comprehensive LisH protein interaction network and uncover a regulatory axis involving Lis1-ARIH2 that is important for dynein dependent intracellular transport.

## Highlights

• A comprehensive interaction network of 27 human LisH domain proteins is established
• Lis1, a LisH protein, associates with CSN to facilitate the deneddylation of ARIH2
• Neddylation of ARIH2 activates its ubiquitin ligase activity
• ARIH2 ubiquitinates the DIC1 subunit of the dynein complex and promotes dynein function

## Graphical abstract

**Figure:**
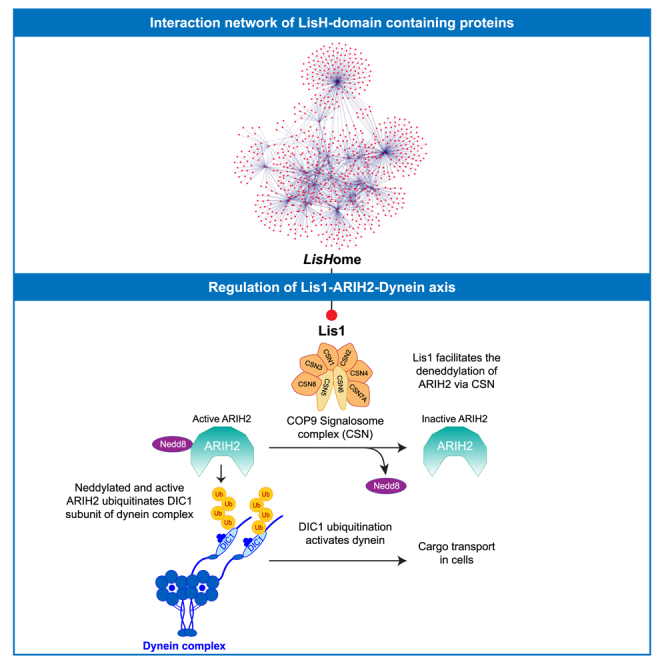

## Introduction

Protein-protein interactions play a crucial role in maintaining cellular homeostasis. Protein-protein interactions are mediated by conserved domains such as WD repeats, Ankyrin domains, Kelch repeats, etc., which impart a specific function to a protein and are essential for all the cellular processes. LisH domain was initially discovered in Lis1 as a stretch of over 34 amino acids at the N-terminus responsible for protein dimerization.^1^ Later, a similar motif was found in multiple intracellular proteins, such as katanin p60 subunits, muskelin, Nopp140, etc. and was termed Lis1-Homology (LisH) domain.^2^ The deletion of LisH domain in proteins such as Lis1, TBL1X, and OFD1 led to defects in protein oligomerization and proper cellular localization.^3^ Further, mutations in several conserved residues in the LisH domain of these proteins led to significant loss in the protein half-life.^3^ Furthermore, LisH-domains of KATNAL2 in *Tetrahymena*,^4^ in muskelin (MKLN1),^5^ and in the fibroblast growth factor receptor 1 oncogene partner (FGFR1OP),^6^ is essential for subcellular localization, dimerization, and stability of the protein. The oligomerization property of the LisH domain is also essential to form functional oligomers of multi-protein complexes, such as the dimerization of the LisH domain in DCAF1 promotes the dimerization of CRL4^DCAF1^, which enhances the substrate ubiquitination.^7^ Additionally, the dimerization of the LisH domain in SMU1 promotes the oligomerization of the SMU1-RED complex and increases the sites for additional protein-protein interactions during splicing.^8^ In addition to its dimerization property, LisH domain proteins also provide sensitivity and specificity to the multi-protein complexes they are associated with. Loss of the LisH domains of DCAF1 or SMU1 led to the disassembly of their corresponding E3 ligase complexes and their substrates.^9^^,^^10^

While LisH-domain-containing proteins are involved in multiple cellular functions via interacting with specific proteins, the global interactome map of human LisH domain containing proteins is unavailable. We performed a global search for LisH domain and retrieved 28 human proteins that contain the LisH domain. After purifying the interacting partners of each protein, we established an interaction network of LisH-domain containing proteins. To further characterize the interaction network, we studied the functional enrichment of protein complexes associated with LisH-domain-containing proteins in biological processes and signaling pathways, and their associations with multi-protein complexes, disease-linked pathways, and cancer. To validate the functional relevance of these interacting partners, we used Lis1 as an example and characterized its association with its interacting partners identified in our mass spectrometry dataset.

Lis1 is a LisH-domain containing protein, which is a known regulator of a molecular motor, dynein.^11^^,^^12^ Lis1 has been implicated in the regulation of dynein by not only stalling it on microtubules and restricting its motility,^13^^,^^14^ but also assisting it in localizing to microtubules and forming active dynein complexes.^15^^,^^16^ Being a multifunctional regulator that has been associated with several steps of the regulation of dynein activation and inactivation, it is important to understand the interacting partners of Lis1 to get a deeper understanding of the mechanism in cells. The regulation of dynein by Lis1 has been well studied in diverse systems ranging from single molecule *in vitro* setups to yeast, neurons, and mammalian systems; however, the precise mechanism behind the critical regulation is unknown. Here, we identified an RBR-type E3 ubiquitin ligase ARIH2 as an important regulatory player in Lis1 mediated dynein-based transport in cells.

## Results

### Establishing an interactome of LisH-domain containing proteins (*LisH*ome) in humans

**Figure 1:**
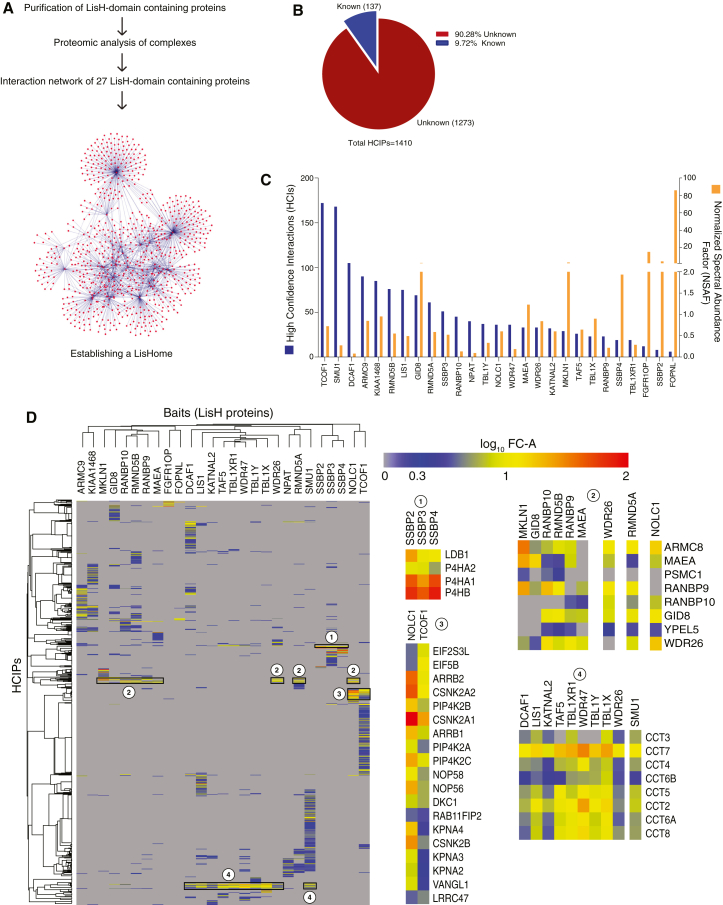
Analysis of human LisH domain protein interactome (A) A step-by-step diagrammatic representation of establishing a protein interaction network of LisH-domain containing proteins. (B) High confidence interactions mediated by LisH proteins and the interacting proteins were compared with known interactions, and the distribution of known and unknown interactions was plotted. (C) Expression of each LisH protein (represented by normalized spectral abundance factor (NSAF) for each bait, orange) and HCIs (blue) found in respective bait purification was plotted in a clustered graph. (D) Heatmap generated from hierarchical clustering of the 771 HCIPs for 27 LisH proteins. The color of the interacting protein corresponds to its log_10_ of the fold change (FC-A) value.

To identify LisH-domain containing proteins, we performed a global search for the LisH domain (ID: PS50896) using the UniProt database. We retrieved 28 human proteins that contain LisH domain of which nine exhibited a combination of LisH domain with WD repeats in their architecture (Figure S1A; Table S1). We established a phylogenetic tree of LisH-domain containing proteins based on their sequence similarities to represent the evolutionary relationships between different proteins within this family (Figure S1B). LisH-domain-containing proteins are found in various cellular compartments, based on localization data from the UniProt database (Figure S1C). We cloned 27 LisH-domain-containing proteins into a triple tagged (SBP-Flag-S) Gateway-compatible vector. We purified each LisH-domain containing protein using a biochemical approach of tandem-affinity purification followed by mass spectrometry analysis (TAP-MS), to understand their associated protein complexes. Each protein was individually expressed in HEK293T cells, and protein complexes were isolated through two rounds of affinity purification using streptavidin-binding protein and S protein tags (Figures S2A–S2J). The interacting proteins were identified via LC-MS/MS analysis. TAP-MS of LisH-domain containing proteins has established an interactome (also known as *LisH*ome here), which identified several uncharacterized associations in cells (Figure 1A). A total of 20,287 interactions were obtained from 27 LisH protein purifications. To filter out the nonspecific interactors, we used the CRAPome^17^ tool to compare our dataset against control SFB, control GFP, and eight other CRAPome control purifications. Interactome data were filtered with different scores such as empirical fold-change scores (FCA and FCB), SAINT score (SS), and interaction specificity score (IS). By using a SAINT score cut off of 0.7, FCA ≥1.8, FCB ≥2, and IS > 1, we identified 1410 high-confidence interactions (HCIs), mediated by 771 high-confidence interacting proteins (HCIPs) (Table S2). Out of 1410 HCIs, around 1273 interactions (90% of interactions) are unknown (Figure 1B). Although the expression of the SFB-tagged LisH proteins varied over a wide range, the number of HCIs did not correlate with the normalized spectral abundance of each bait, thus highlighting the unbiased nature of the list of interactors identified in our study (Figure 1C; Table S3). A correlation would be expected if overexpression leads to non-physiological interactions, which is clearly not the case in this study. Moreover, analysis of HCIPs that cluster together for an individual LisH protein can reveal the proteins shared by multiple baits and the proteins that participate in a complex (Table S4), as seen in the zoomed in clusters (Figure 1D). Together, we established an interactome dataset for human LisH-domain containing proteins.

### Association of LisH-domain containing proteins with signaling pathways and cellular processes

**Figure 2:**
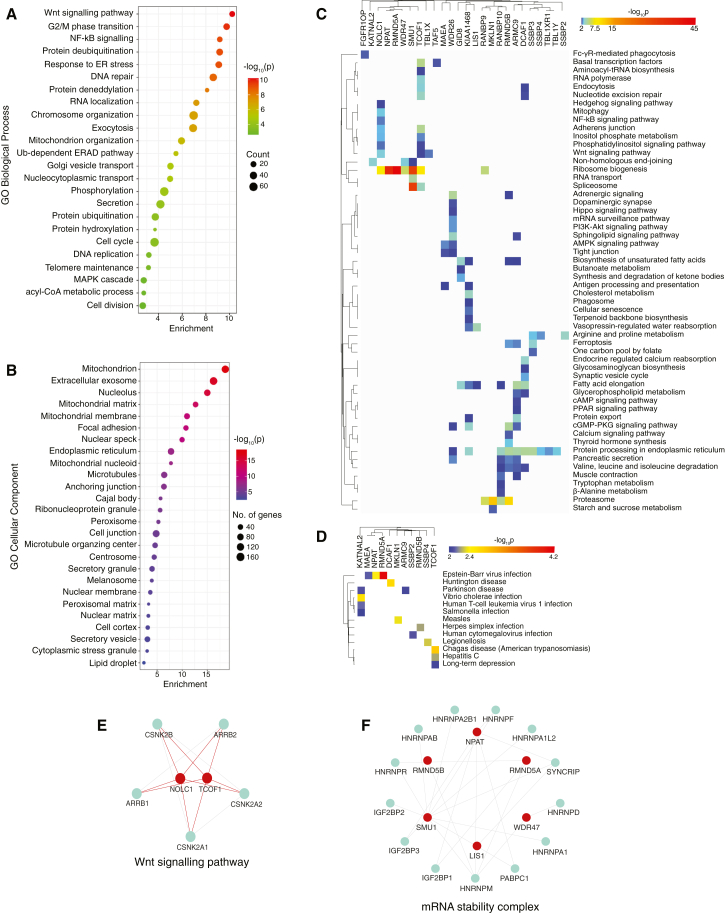
Association of LisH domain containing proteins with pathways and cellular processes (A) Interactors were analyzed by ShinyGO and their GO distribution into various functional biological processes. (B) subcellular localization was shown. (C) KEGG pathway mapping was performed using the interaction dataset, and the pathway enrichment was plotted for each LisH protein. Enrichment *p*-values were represented on the heatmap. (D) Diseases enrichment is represented on the heatmap cluster for each LisH protein. (E) Interaction network of LisH proteins and the proteins associated with Wnt signaling. (F) mRNA stability complex. The circles in red are the LisH proteins, while the circles in green are the interactors.

The functional classification of HCIPs using ShinyGO^18^ revealed that *LisH*ome interactors are broadly distributed across diverse biological processes (Figure 2A) and exhibit varied cellular localizations (Figure 2B; Table S5). This highlights the involvement of human LisH proteins in a wide range of biological functions. To further understand the functional role of these interactions, we annotated them to KEGG pathways using ConsensusPathDB^19^ (Table S6). Importantly, several key cellular signaling pathways, such as PI3K-Akt, Hippo, Wnt, Hedgehog, and several metabolic pathways, were highly enriched for HCIPs of different LisH proteins. Some key cellular processes such as endocytosis, DNA repair, splicing machinery, protein degradation processes are also enriched in the HCIPs of the bait LisH proteins (Figure 2C). Moreover, we also found several diseases such as Parkinson’s disease, Huntington disease, Epstein-Barr virus infection etc getting enriched in the interacting partners of some of the LisH proteins (Figure 2D).

We found several known as well as uncharacterized associations of LisH-domain containing proteins enriched with various cellular signaling pathways. For example, we found the known associations of NOLC1 and TCOF1 with Wnt signaling pathway components (Figure 2E), and SMU1 and TCOF1 with eukaryotic initiation factor complex components (Figure S3A). Whereas, we also found previously uncharacterized associations of several other LisH domain containing proteins to be associated with protein folding complexes such as CCT complex (Figure S3B), Prefoldin complex (Figure S3C), as well as mRNA stability complex (Figure 2F). The CCT complex is a molecular chaperone essential for the folding of diverse proteins, with many of its known substrates being WD40-repeat proteins.^20^^,^^21^ Since several LisH proteins also contain WD40 repeats, it is plausible that they may serve as substrates of the CCT complex. Moreover, the Prefoldin complex assists in protein folding by binding to unfolded proteins and delivering them to CCT.^22^ Thus, LisH proteins that show specific enrichment with CCT and prefoldin complexes may rely on these machineries for proper folding and oligomerization. In conclusion, our enrichment analysis not only confirmed known functions but also connected previously unexplored LisH-domain-containing proteins to specific biological processes and cellular signaling pathways.

### Association of LisH-domain containing proteins with multi-protein complexes

One of the main advantages of affinity purification is the ability to capture multi-protein complexes associated with baits. The association of a LisH protein with a specific protein complex may suggest its role in a specific pathway. We next used COMPLEAT,^23^ a protein complex enrichment analysis tool, to find out protein complexes associated with individual LisH protein baits (Table S7). We found some known multi-protein complexes associated with the LisH proteins, hence validating our analysis. For example, several LisH proteins acting together as CTLH E3 ligase complex^24^^,^^25^ (Figure S3D), RMND5B, being a RING E3 ligase, is involved with proteasome complex^24^^,^^26^ and Npl4-Ufd1-VCP complex^27^ (Figures S3E and S3F), and TCOF1 and NOLC1, while localizing in nucleolus can function together in ribonucleoprotein biogenesis with boxC/D snoRNP complex^28^^,^^29^ (Figure S3G), and also for trafficking of cellular messengers via PIP kinase complex^30^^,^^31^ (Figure S3H).

**Figure 3:**
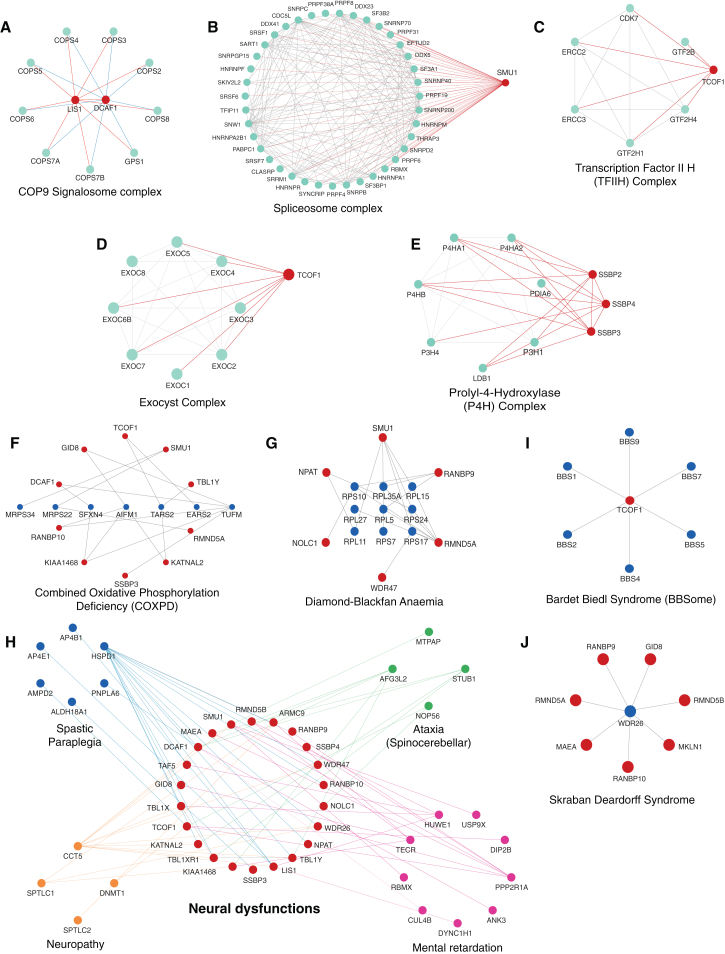
Association of LisH domain containing proteins with multiprotein complexes and disease-linked genes The interactors from each LisH-domain containing protein were searched against the COMPLEAT database and the representative LisH protein−multiprotein complexes, such as (A) COP9 signalosome complex. (B) Spliceosome complex. (C) Transcription factor II H complex. (D) Exocyst complex. (E) Prolyl-4-hydroxylase (P4H) complex, are shown. Interaction network of LisH proteins with genetic diseases such as (F) combined oxidative phosphorylation deficiency (COXPD). (G) Diamond Blackfan anemia. (H) neural dysfunctions. (I) Bardet-Biedl syndrome. (J) Skraban Deardorff syndrome (from the OMIM database), built by Cytoscape, is shown. The circles in red are the LisH proteins, while circles in green/blue are the interactors.

On the other hand, we also found many previously unknown interactions of LisH proteins with multiprotein complexes. For instance, the interaction of Lis1 and DCAF1 with COP9 signalosome complex (Figure 3A), the interaction of SMU1 with spliceosome complex (Figure 3B), interaction of TCOF1 with transcription factor II H complex (Figure 3C) as well as with exocyst complex (Figure 3D), and paralogs such as SSBP2, SSBP3, and SSBP4 with components of Prolyl-4-Hydrolase (P4H) complex (Figure 3E), were identified in our study. Thus, interactions of LisH proteins with multiprotein complexes may uncover previously unidentified functions of LisH-domain-containing proteins.

### Linking *LisH*ome with disease phenotypes

A plethora of studies have identified mutations and deletions in genomic loci of LisH proteins associated with various human diseases.^2^^,^^32^^,^^33^^,^^34^ For example, Lis1 plays a role in lissencephaly, TCOF1 is associated with Treacher Collins Syndrome (TCS), and TBL1X participates in Ocular Albinism with late-onset sensorineural deafness (OASD). Although LisH proteins are linked to various diseases, the mechanisms by which these alterations contribute to disease development remain unclear. To explore the role of LisH-domain-containing proteins in diseases and identify biochemically related proteins linked to specific disease phenotypes, we integrated data on altered genomic loci with the LisH protein interaction network. Using OMIM-annotated disease-linked genes,^35^ we analyzed their interactions with LisH proteins to uncover potential connections to disease mechanisms. We identified 201 disease-linked proteins that interact with 27 LisH-domain containing proteins (Table S8). We found multiple LisH proteins associated with clusters of similar diseases such as congenital disorder of glycosylation (CDG) (Figure S3I), combined oxidative phosphorylation deficiency (COXPD) (Figure 3F), and Diamond Blackfan Anemia (Figure 3G), and neural dysfunctions (Figure 3H) such as spastic paraplegia, spinocerebellar ataxia, etc.

On the other hand, we also found a single LisH protein connected with rare genetic disorders with severe growth retardation and multiple organ dysfunction. We found SMU1 to be associated with 3M syndrome (Figure S3J), and TCOF1 to be associated with BBSome complex responsible for Bardet Biedl syndrome (Figure 3I). LisH proteins, which are known to form CTLH E3 ligase complex, are enriched in Skraban Deardorff syndrome (Figure 3J), also known as WDR26-related intellectual disability disorder, characterized by developmental delays, seizures, intellectual disability, and neurological symptoms. This data suggests the presence of multiple levels of LisH protein-dependent regulation in these diseases, highlighting the potential complexity of their involvement in disease phenotypes.

We further matched the LisH interactome to the COSMIC (Cancer Gene Census) dataset,^36^ which catalogs genes mutated in human cancers. Of the 27 LisH proteins analyzed, 20 were associated with cancer-linked proteins (Table S9). In total, we identified 50 interactors within the LisH interactome genetically linked to various tumor types, resulting in 80 interactions overall (Figure S3L). We identified both known and unknown LisH protein interactions with cancer-associated proteins. For instance, the TCOF1 and Lis1 are found to be associated with cancer-associated proteins (Figures S3M and S3N), functioning as either oncogenes or tumor suppressors. As many of these interactions between LisH proteins and disease-linked proteins remain unexplored, our LisH protein-disease interactome provides a valuable resource for identifying different pathways and mechanisms regulated by LisH-domain-containing proteins in disease development.

### ARIH2 associates with Lis1 and COP9 signalosome (CSN)

**Figure 4:**
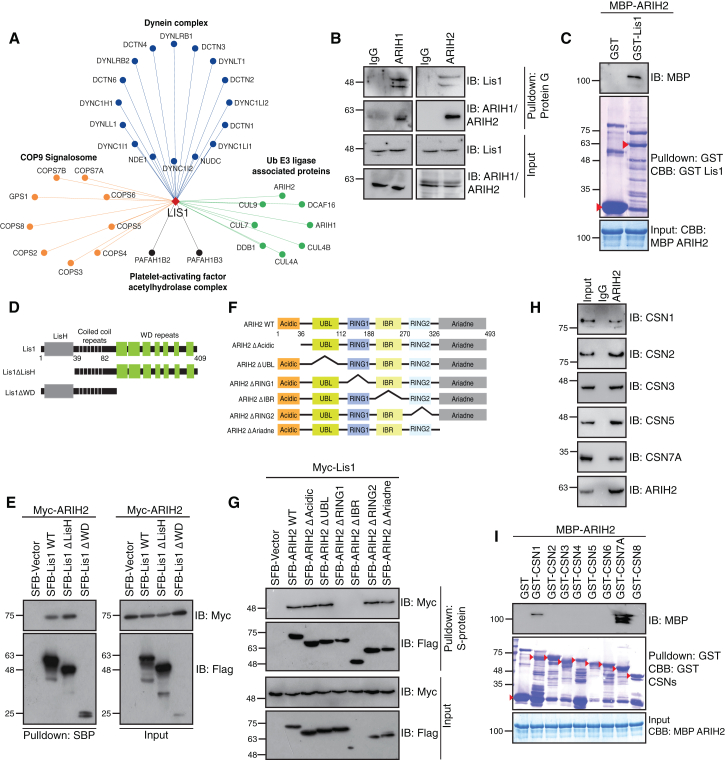
ARIH2 associates with Lis1 and CSN (A) Interaction network of Lis1 with its associated proteins and complexes (COP9 Signalosome and ubiquitin E3 ligases) derived from mass spectrometry analysis is shown. (B) HEK293T cell lysates were subjected to immunoprecipitation with either IgG or ARIH1 or ARIH2. Presence of Lis1 in the immunoprecipitants of ARIH1 or ARIH2 was detected by western blotting with respective antibodies. (C) Bacterially purified recombinant GST-Lis1 was incubated with purified MBP-ARIH2. The eluates were immunoblotted with the MBP antibody. The purified proteins are shown with a red arrow. CBB: Coomassie Brilliant Blue. (D) Pictorial representation of the domain architecture of Lis1 protein is shown. (E) HEK 293T cells were transfected with SFB Lis1 WT, SFB ΔLisH, and SFB Lis1 ΔWD along with Myc ARIH2 plasmids. Cells were lysed and incubated with S-protein beads. Interaction was detected by immunoblotting with Myc antibody. (F) Pictorial representation of the domain architecture of ARIH2 protein is shown. (G) HEK 293T cells were transfected with SFB ARIH2 WT, SFB ARIH2 ΔAcidic, SFB ARIH2 ΔUBL, SFB ARIH2 ΔRING1, SFB ARIH2 ΔIBR, SFB ARIH2 ΔRING2, and SFB ARIH2 ΔAriadne along with Myc Lis1 plasmids. Cells were lysed and incubated with S-protein beads. Interaction was detected by immunoblotting with Myc antibody. (H) HEK293T cell lysates were subjected to immunoprecipitation with either IgG or ARIH2. Presence of CSN subunits in the immunoprecipitants of ARIH2 was detected by Western blotting with respective antibodies. (I) Bacterially purified recombinant GST-CSN subunits were incubated with purified MBP-ARIH2. The eluates were immunoblotted with the MBP antibody. The purified proteins are shown with a red arrow. CBB: Coomassie Brilliant Blue.

Subsequently, to validate the functional importance of this network, in this study, we characterized the association of ARIH2 with Lis1. We found Cullin components (Cul4A/B, Cul7, Cul9, and DDB1) and RBR E3 ligases (ARIH1 and ARIH2) as Lis1 associated interactors, along with the known interactors such as dynein, dynactin, Nde1, and PAF acetyl hydrolase components (Figure 4A). Interestingly, COP9 Signalosome (CSN), a conserved multi-subunit deneddylase complex^37^ and a well-known regulator of Cullin E3 ligases^38^ enriched in the Lis1 proteome. Their association with the Lis1 or dynein pathway so far is unknown. We confirmed the association of Cul4A, Cul7, DDB1, ARIH1, ARIH2, and CSN complex subunits with Lis1 using exogenously expressed proteins in HEK 293T cells (Figures S4A–S4D). While Cullin's association with CSN is well established, the connection between RBR E3 ligases and CSN is unknown. Therefore, we further focused on understanding the interplay between Lis1-CSN-ARIH connections. We validated the endogenous association of Lis1 with ARIH1 and ARIH2 in cells (Figure 4B). *In vitro* pulldown assay using bacterially purified recombinant proteins suggested that Lis1 directly binds with ARIH2 (Figure 4C). Lis1 comprises of a LisH domain at its N-terminus, followed by a short stretch of coiled-coil repeats, and a set of seven WD repeats spanning till the C-terminus of the protein (Figure 4D). We made Lis1 deletion mutants (ΔLisH or ΔWD) and tested their interaction with ARIH2 to identify the ARIH2 binding domain on Lis1. The deletion of WD repeats significantly reduced the Lis1 binding with ARIH2 in cells, suggesting that Lis1 binds to ARIH2 with its WD repeats (Figure 4E). Further, to map the binding region of Lis1 on ARIH2, we made ARIH2 deletion constructs of individual domains (ΔAcidic, ΔUBL, ΔRING1, ΔIBR, ΔRING2, and ΔAriadne) (Figure 4F). The deletion of either RING1 or IBR reduced the binding of ARIH2 with Lis1, suggesting that both the regions on ARIH2 are essential for binding with Lis1 (Figure 4G). This data suggests that ARIH2 is an interactor of Lis1.

As Lis1 and CSN exist together,^39^ we next tested if ARIH2 associates with CSN too. Along with Lis1, ARIH2 also interacts with CSN components in cells (Figure S4E). Also, we validated the endogenous association of ARIH2 with CSN complex subunits (Figure 4H). To understand if ARIH2 directly binds with CSN, we used bacterially purified recombinant proteins. The *in vitro* pulldown assay revealed that ARIH2 specifically binds with CSN1 and CSN7A subunits (Figure 4I). Taken together, our data suggest that ARIH2 interacts with Lis1 and CSN.

### COP9 signalosome deneddylates ARIH2

**Figure 5:**
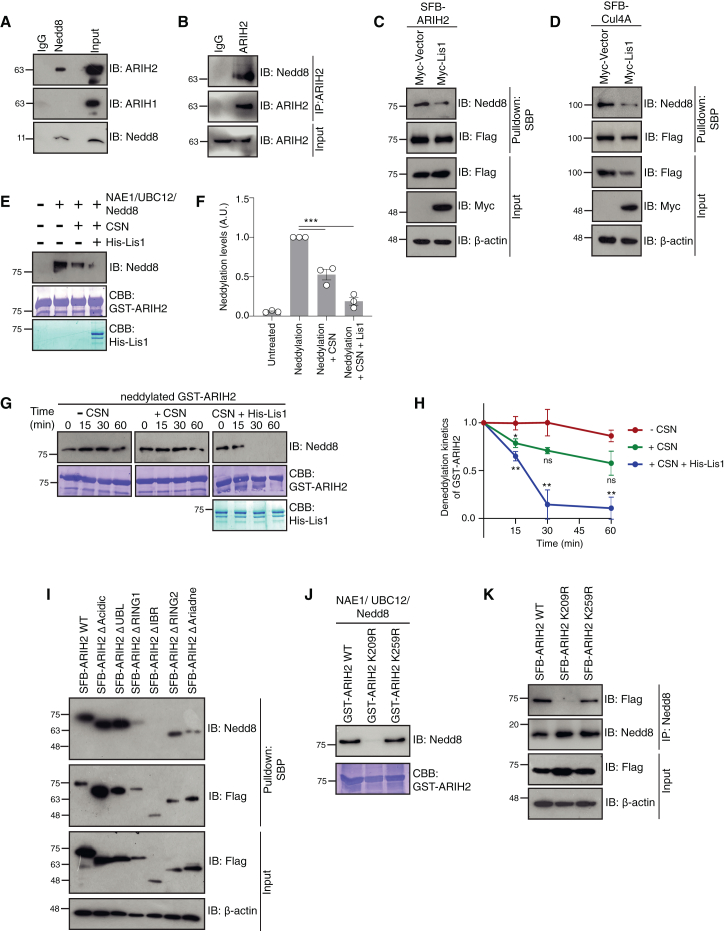
ARIH2 gets neddylated and is a deneddylation substrate of CSN (A) HEK293T cell lysates were subjected to denaturing immunoprecipitation with either IgG or Nedd8. Presence of ARIH1 or ARIH2 in the immunoprecipitants of Nedd8 was detected by Western blotting with respective antibodies. (B) HEK293T cell lysates were subjected to denaturing immunoprecipitation with either IgG or ARIH2. Presence of Nedd8 in the immunoprecipitants of ARIH2 was detected by western blotting with respective antibodies. (C) HEK 293T cells were transfected with SFB-ARIH2 along with Myc-Lis1 plasmids. Cells were then lysed under denaturing conditions and incubated with streptavidin beads. Neddylated protein was detected by immunoblotting with anti-Nedd8 antibody. (D) HEK 293T cells were transfected with SFB-Cul4A, along with Myc-Lis1 plasmids. Cells were then lysed under denaturing conditions and incubated with streptavidin beads. Neddylated protein was detected by immunoblotting with anti-Nedd8 antibody. (E) Bacterially purified GST-ARIH2 was incubated with NAE1, UBC12, and Nedd8 for *in vitro* neddylation for 60 min, and with purified CSN and His-Lis1 for *in vitro* deneddylation for 60 min. Neddylated protein was visualized by immunoblotting with anti-Nedd8 antibody. CBB: Coomassie Brilliant Blue. (F) Quantification for the experiment shown in (E). Data are represented as mean ± SEM from three biological replicates (∗∗∗*p* < 0.0001; one-way ANOVA followed by Dunnett’s multiple comparisons test). (G) Bacterially purified GST-ARIH2 was incubated with NAE1, UBC12 and Nedd8 for *in vitro* neddylation for 60 min, and with purified CSN in combination with His-Lis1 for *in vitro* deneddylation for the indicated time durations. Neddylated protein was visualized by immunoblotting with anti-Nedd8 antibody. (H) Quantification for the experiment shown in (G). Data are represented as mean ± SEM from three biological replicates (∗*p* = 0.0291, ∗∗*p* = 0.0036, ns = not significant; two-way ANOVA followed by Dunnett’s multiple comparisons test). (I) HEK 293T cells were transfected with SFB ARIH2 WT, SFB ARIH2 ΔAcidic, SFB ARIH2 ΔUBL, SFB ARIH2 ΔRING1, SFB ARIH2 ΔIBR, SFB ARIH2 ΔRING2, and SFB ARIH2 ΔAriadne plasmids. Cells were then lysed under denaturing conditions and incubated with streptavidin beads. Neddylated protein was detected by immunoblotting with anti-Nedd8 antibody. (J) Bacterially purified GST-ARIH2 WT, GST-ARIH2 K209R, and GST-ARIH2 K259R were incubated with NAE1, UBC12, and Nedd8 for *in vitro* neddylation for 60 min. Neddylated protein was visualized by immunoblotting with anti-Nedd8 antibody. CBB: Coomassie Brilliant Blue. (K) HEK 293T cells were transfected with SFB ARIH2 WT, SFB ARIH2 K209R, and SFB ARIH2 K259R plasmids. Cells were then lysed under denaturing conditions and subjected to immunoprecipitation with Nedd8 antibody. Neddylated protein was detected by immunoblotting with anti-Flag antibody.

Studies have shown that ARIH2 associates specifically with Cul5 to form multi-protein complexes and for the activation of its ubiquitin ligase activity.^40^^,^^41^^,^^42^ Since we did not get Cul5 in the Lis1 interactome, we hypothesized that ARIH2 may have additional routes of activation other than Cul5. As CSN is a deneddylating enzyme, we next tested if ARIH2 is a substrate of CSN. Firstly, we tested if ARIH proteins undergo neddylation. Immunoprecipitation using Nedd8-specific antibody suggested that ARIH2 is specifically neddylated in cells (Figure 5A). Immunoprecipitation using endogenous ARIH2 followed by immunoblotting with Nedd8-specific antibody further confirmed that ARIH2 gets neddylated (Figure 5B). Moreover, this neddylation gets significantly reduced in the presence of Lis1, which was also observed in Cul4A, a known neddylation substrate (Figures 5C and 5D). Subsequently, an *in vitro* neddylation and deneddylation assay on purified GST-ARIH2 indicated that CSN-mediated deneddylation on ARIH2 is significantly higher in the presence of purified Lis1 (Figures 5E and 5F). Next, a time dependent deneddylation assay on GST-ARIH2 by CSN with or without Lis1, revealed that Lis1 enhances the deneddylation kinetics of CSN on ARIH2 (Figures 5G and 5H). Interestingly, Lis1 mediated deneddylation kinetics on ARIH2 WT were faster than ARIH2 ΔRING1 (Figures S4F and S4G), further confirming the significance of Lis1 binding on RING1 and IBR domains of ARIH2. Additionally, we sought to find the site of neddylation on ARIH2. Neddylation levels on domain-deletion mutants of ARIH2 revealed that the IBR domain of ARIH2 contains the potential neddylation site (Figure 5I). The IBR domain contains two lysine residues – K209 and K259 – which we mutated to arginine residues and tested for the loss of neddylation. We saw a significant decrease in the neddylation levels with the K209R mutant of ARIH2 (Figures 5J and 5K), suggesting that K209 is the neddylation site on ARIH2. Together, this suggests that ARIH2 gets neddylated and Lis1 promotes its deneddylation via CSN.

### Neddylation on ARIH2 promotes its ubiquitin ligase activity

**Figure 6:**
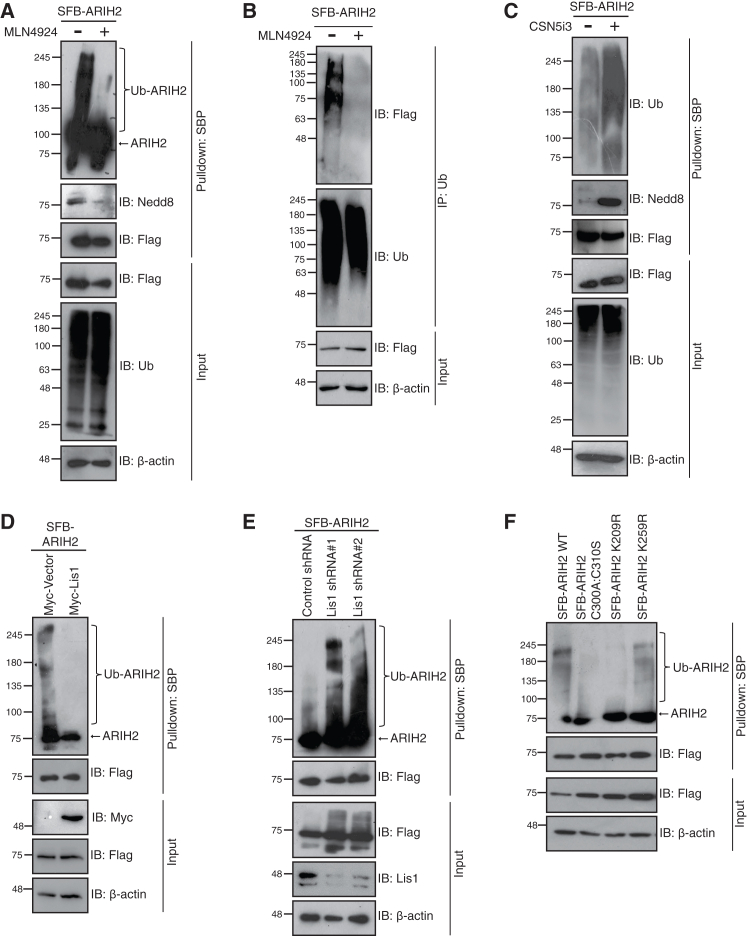
ARIH2 neddylation promotes its ubiquitin ligase activity (A) HEK293T cells were transfected with SFB-ARIH2. Cells were treated with 3 μM MLN4924 for 4 h before harvesting. Cell lysates were subjected to denaturing immunoprecipitation and incubated with streptavidin beads. Presence of ubiquitination and neddylation was detected by Western blotting with respective antibodies. (B) HEK293T cells were transfected with SFB-ARIH2. Cells were treated with 3 μM MLN4924 for 4 h before harvesting. Cell lysates were subjected to denaturing immunoprecipitation and immunoprecipitated with Ub antibody. Ubiquitinated protein was detected by immunoblotting with anti-Flag antibody. (C) HEK293T cells were transfected with SFB-ARIH2. Cells were treated with 3 μM CSN5i3 for 4 h before harvesting. Cell lysates were subjected to denaturing immunoprecipitation and incubated with streptavidin beads. Presence of ubiquitination and neddylation was detected by Western blotting with respective antibodies. (D) HEK293T cells were transfected with SFB-ARIH2 along with Myc Lis1 plasmids. Cell lysates were subjected to denaturing immunoprecipitation and incubated with streptavidin beads. Presence of ubiquitination was detected by Western blotting with respective antibodies. (E) Control and Lis1 depleted HEK293T cell lysates were transfected with the SFB-ARIH2 plasmid. Cell lysates were subjected to denaturing immunoprecipitation and incubated with streptavidin beads. Presence of ubiquitination was detected by Western blotting with respective antibodies. (F) HEK293T cells were transfected with SFB-ARIH2 WT, SFB-ARIH2 C300A:C310S, SFB-ARIH2 K209R, and SFB ARIH2 K259R plasmids. Cell lysates were subjected to denaturing immunoprecipitation and incubated with streptavidin beads. Presence of ubiquitination was detected by Western blotting with respective antibodies.

Ubiquitin E3 ligases undergo a process of auto-ubiquitination to regulate stability and activity of themselves.^43^^,^^44^ It is also seen as a general mechanism for the activation of the ubiquitin ligases.^45^ To understand how neddylation influences the ligase activity of ARIH2, we studied the auto-ubiquitination of ARIH2 in the presence of a neddylation inhibitor (MLN4924). Perturbing the neddylation decreased the auto-ubiquitination activity of ARIH2 (Figures 6A and 6B), while blocking the deneddylation activity of CSN (using CSN5i3) increased the auto-ubiquitination of ARIH2 (Figure 6C), suggesting that neddylation is essential for the ubiquitin ligase activity of ARIH2. As Lis1 enhanced the deneddylation on ARIH2, we tested if it also plays a role in the autoubiquitination of ARIH2. Interestingly, the exogenous expression of Lis1 reduced the ARIH2 autoubiquitination (Figure 6D), while knockdown of Lis1 increased the ubiquitination levels (Figure 6E), similar to the pattern observed for neddylation. Further, ARIH2 auto-ubiquitination was also reduced in the neddylation defective mutant K209R (Figure 6F). Together, these data suggest that ARIH2 neddylation promotes its ubiquitin ligase activity.

### ARIH2 ubiquitinates dynein intermediate chain 1

**Figure 7:**
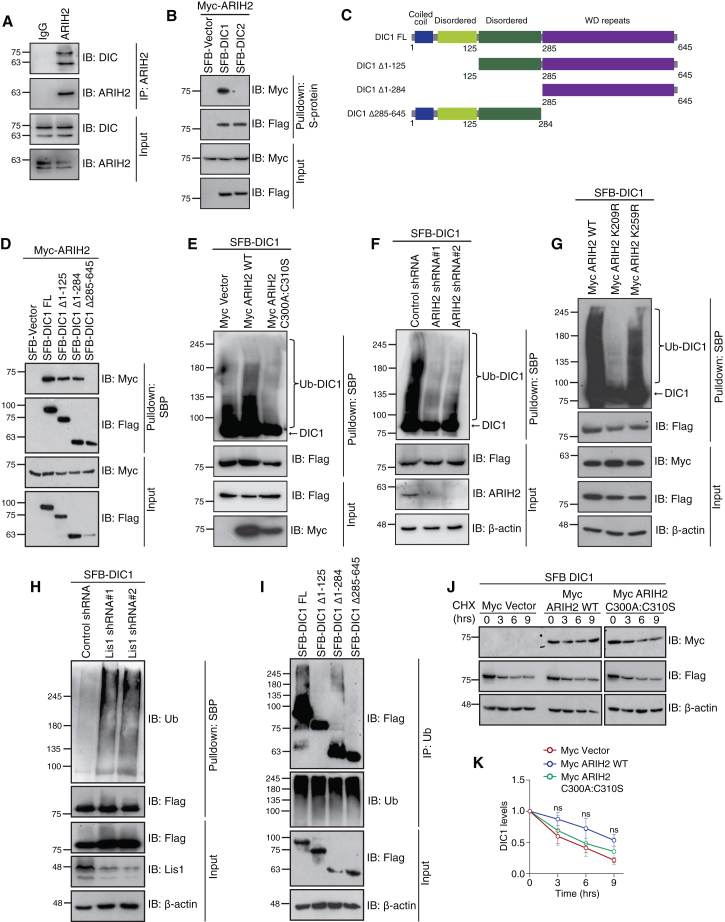
ARIH2 ubiquitinates DIC1 (A) HEK293T cells were subjected to immunoprecipitation with either IgG or ARIH2. Presence of DIC in ARIH2 immunoprecipitants was detected by Western blotting with respective antibodies. (B) HEK 293T cells were transfected with the dynein subunits SFB-DIC1 and SFB-DIC2 along with Myc-ARIH2 plasmids. Cells were lysed and incubated with S-protein beads. Interaction was detected by immunoblotting with Myc antibody. (C) Pictorial representation of the domain architecture of DIC1 protein is shown. (D) HEK 293T cells were transfected with SFB DIC1 WT, SFB DIC1 Δ1-125, SFB DIC1 Δ1-284, and SFB DIC1 Δ285-645, along with Myc ARIH2 plasmids. Cells were lysed and incubated with S-protein beads. Interaction was detected by immunoblotting with Myc antibody. (E) HEK293T cells were transfected with SFB-DIC1 along with Myc-ARIH2 WT and Myc-ARIH2 C300A:C310S plasmids. Cell lysates were subjected to denaturing immunoprecipitation and incubated with streptavidin beads. Presence of ubiquitination was detected by Western blotting with respective antibodies. (F) Control and ARIH2 depleted HEK293T cells were transfected with the SFB-DIC1 plasmid. Cell lysates were subjected to denaturing immunoprecipitation and incubated with streptavidin beads. Presence of ubiquitination was detected by Western blotting with respective antibodies. (G) HEK293T cells were transfected with SFB-DIC1 along with Myc-ARIH2 WT, Myc-ARIH2 K209R, and Myc-ARIH2 K259R plasmids. Cell lysates were subjected to denaturing immunoprecipitation and incubated with streptavidin beads. Presence of ubiquitination was detected by Western blotting with respective antibodies. (H) Control and Lis1 depleted HEK293T cell lysates were transfected with the SFB-DIC1 plasmid. Cell lysates were subjected to denaturing immunoprecipitation and incubated with streptavidin beads. Presence of ubiquitination was detected by western blotting with respective antibodies. (I) HEK 293T cells were transfected with SFB DIC1 WT, SFB DIC1 Δ1-125, SFB DIC1 Δ1-284, and SFB DIC1 Δ285-645 plasmids. Cell lysates were subjected to denaturing immunoprecipitation and immunoprecipitated with Ub antibody. Ubiquitinated protein was detected by immunoblotting with anti-Flag antibody. (J) HEK293T cells were transfected with SFB-DIC1 along with either Myc-Vector, Myc-ARIH2 WT, and Myc-ARIH2 C300A:C310S plasmids. 24 h after transfection, cycloheximide (CHX) was added at 50 μg/ml and chased until the indicated time points. Protein levels were detected by immunoblotting with respective antibodies. (K) Quantification for the experiment done in (J) through three biological replicates is shown. Data are represented as mean ± SEM (ns: not significant).

Next, we sought to understand the functional significance of the Lis1-ARIH2 connection in cells. Since Lis1 is a bona fide regulator of dynein, we next tested if ARIH2 also associates with dynein. Along with Lis1, ARIH2 also interacts with dynein subunit, dynein intermediate chain, DIC (Figure 7A). Interestingly, while DIC1 and DIC2 are orthologs in humans with a 76% similarity (Figure S5A), ARIH2 specifically associates with DIC1 (Figure 7B). To understand ARIH2’s preference for DIC1 over DIC2, we used the structural information of these interactors. We used Alphafold2 to model the structures of ARIH2, DIC1, and DIC2, and then docked ARIH2 on DIC1 and DIC2 using HDOCK. The resulting models of individual proteins are strikingly similar to their known structures and biophysical properties. Whereas the majority of ARIH2 were α-helices, half of DIC1 and DIC2 were unstructured, followed by a β propeller in the C-termini. The docked models of ARIH2:DIC1 and ARIH2:DIC2 revealed a difference in the contact sites shared by ARIH2 with DIC1 and DIC2 (Figures S5B and S5C) and a lower score of binding affinity with DIC2 (Figure S5D). This validated the biochemical association and preference of ARIH2 for DIC1 over DIC2. The structural model of ARIH2:DIC1 also revealed that DIC1 docks on ARIH2 via its C-terminal WD repeats, which was also validated biochemically using DIC1 truncation mutants (Figure 7C), suggesting that WD repeats are the binding interface for ARIH2 on DIC1 (Figure 7D).

Next, we found that ARIH2 enhances the ubiquitination of DIC1 in cells via its catalytic ligase activity (Figures 7E and S6A). The depletion of ARIH2 (Figures 7F and S6B) or using its neddylation defective mutant (Figure 7G) significantly impaired the DIC1 ubiquitination. As Lis1 regulates the ARIH2 deneddylation, the depletion of Lis1 increases the DIC1 ubiquitination (Figure 7H), which further confirms that neddylated ARIH2 ubiquitinates DIC1. This suggests that DIC1 is a substrate of ARIH2. Furthermore, the loss of ARIH2 binding region of DIC1 (WD repeats) resulted in a significant reduction of ARIH2 mediated ubiquitination of DIC1 (Figure 7I). These data also suggests that although N-terminus and middle region truncated mutants of DIC1 has intact ARIH2 binding, there is a significant reduction in the ubiquitination of these variants, possibly suggesting that there are multiple sites of ubiquitination in these two regions that are being modified by ARIH2.

Ubiquitination is known to regulate protein turnover rates by affecting its cellular levels, half-life, and stability. As ARIH2 is an E3 ligase, we tested the cellular levels of dynein complex components. The steady state levels of overexpressed DIC1, DIC2 (Figure S6C), as well as endogenous Dynein heavy Chain (DHC) and DIC (Figure S6D) remain unchanged in the presence of ARIH2 WT and its ligase dead mutant. ARIH2 is known to associate with Cullins and other E3 ligases,^41^^,^^42^ and interestingly, we also found different E3 ligases in the Lis1 interactome, which suggests a possibility of a cross-talk of multiple ubiquitination pathways around the Lis1-ARIH2 axis, to control dynein complex stability. To understand the effect of another ubiquitination event in the absence of ARIH2, we tested the cellular levels of the dynein complex. The steady state levels of DIC2, light intermediate chains such as LIC1 and LIC2, light chains such as DYNLL1, DYNLRB1, and DYNLT1 remained consistent in the depletion of ARIH2 (Figures S6E–S6J). Additionally, the half-life of DIC1, either in the presence of ARIH2 WT and ligase dead mutant (Figures 7J and 7K), or in the depletion of ARIH2 (Figures S6K and S6L) was unaltered. In fact, increasing concentrations of Lis1—condition, which makes ARIH2 inactive—stabilizes the endogenous DIC levels (Figures S6M and S6N). This rules out the existence of another ubiquitination pathway on Lis1-ARIH2-dynein axis, and suggests that ARIH2 mediated ubiquitination on DIC1 is non-proteolytic and does not affect the cellular levels of dynein complex.

### ARIH2 regulates dynein function

**Figure 8:**
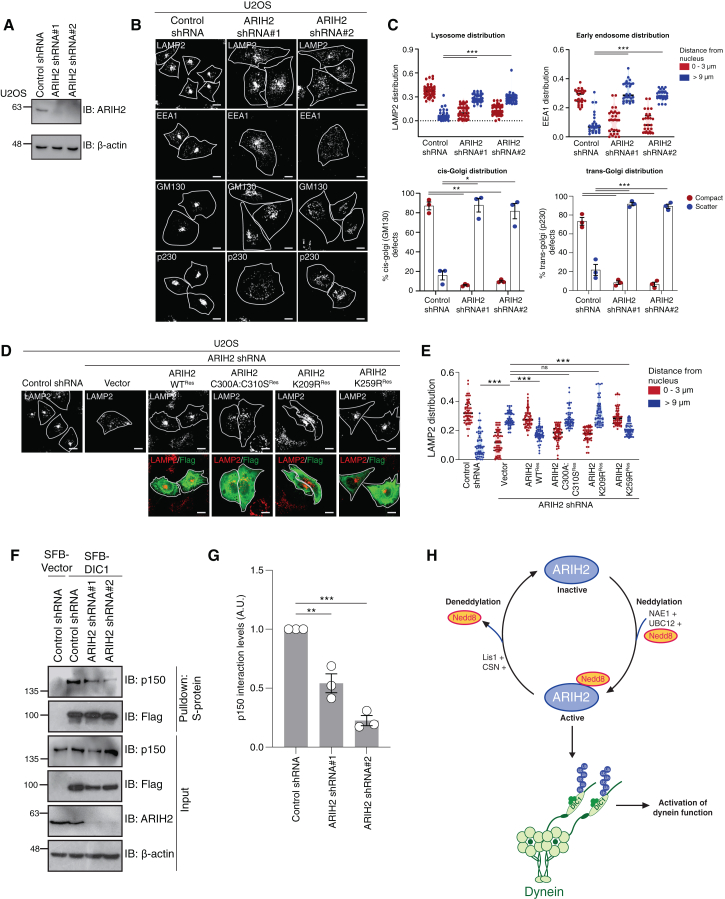
ARIH2 is essential for dynein function (A) U2OS cells were transduced with ARIH2 shRNA, and the knockdown was tested by probing with respective antibodies. (B) Control and ARIH2 depleted stable U2OS cells were fixed and labeled with LAMP2 (lysosomes), EEA1 (early endosome), GM130 (cis-Golgi), and p230 (trans-Golgi) antibodies to visualize their distribution under a confocal microscope. Scale bars: 10 μm. (C) Quantification of the distribution of LAMP2-positive compartments (*n* = 41), EEA1-positive compartments (*n* = 30) in U2OS cells for the experiments shown in (B). Data represent the median ± interquartile range from three independent experiments. (∗∗∗*p* < 0.0001; two-tailed Student’s t test). The golgi distribution has been categorized into two types: compact and scatter. The values plotted are the mean ± SE from three independent experiments. (∗∗*p* < 0.0038; ∗*p* < 0.0393, ∗∗∗*p* < 0.0004; two-way ANOVA followed by Dunnett’s multiple comparisons test). (D) Control and ARIH2 depleted U2OS cells transfected with shRNA resistant SFB ARIH2 WT, SFB ARIH2 C300A:C310S, SFB ARIH2 K209R, and SFB ARIH2 K259R plasmids. The cells were fixed and labeled with LAMP2 (lysosomes) antibody to visualize their distribution under a confocal microscope. Representative images from three independent experiments are shown. Scale bars: 10 μm. (E) Quantification of the distribution of LAMP2-positive compartments (*n* = 56) in U2OS cells for the experiment shown in (D). Data represent are median ± interquartile range from three independent experiments. (∗∗∗*p* < 0.0001; ns = not significant; one-way ANOVA followed by Dunnett’s multiple comparisons test). (F) Control and ARIH2 depleted HEK 293T cells were transfected with the SFB DIC1 plasmid. Cells were lysed and incubated with S-protein beads. Interaction was detected by immunoblotting with p150 antibody. (G) Quantification for the experiment shown in (F). (∗∗*p* = 0.0014, ∗∗∗*p* < 0.0001; one-way ANOVA followed by Dunnett’s multiple comparisons test). The values plotted are the mean ± SE from three independent experiments. (H) The model depicting ARIH2 neddylation enhances its catalytic ligase activity to ubiquitinate dynein intermediate chain 1 and activate dynein function in cells.

Dynein maintains the retrograde movement of endosomes, lysosomes and is essential for maintaining Golgi architecture and dynamics in the cell.^46^^,^^47^ Therefore, to test the role of ARIH2, we next sought to assess the organelle positioning as a readout for dynein activity. As expected, while control cells display predominantly perinuclear positioning of lysosomes, endosomes, and Golgi apparatus due to active dynein, depletion of ARIH2 (Figure 8A) led to the redistribution of lysosomes and endosomes along with Golgi architectural defects (Figures 8B and 8C). This pattern of distribution of organelles phenocopies the depletion of Lis1 and dynein, as shown previously.^39^ Together, our data suggest that ARIH2 ubiquitinates dynein subunit DIC1 and regulates the organelle positioning in cells.

Next, to test if the ubiquitin ligase activity of ARIH2 is required for dynein dependent organelle positioning, we made shRNA-resistant constructs of ARIH2 WT, its catalytically inactive mutant ARIH2 C300A:C310S, neddylation defective mutant K209R, and another lysine mutant K259R within the IBR domain, and expressed them in ARIH2-depleted cells. Wild type ARIH2 and K259R, but not its catalytically inactive mutant or neddylation defective mutant, rescue the lysosomes and endosomes to the perinuclear space and the restoring Golgi architecture (Figures 8D, 8E, and S7A, B), clearly suggesting that the ubiquitin ligase activity of ARIH2 is crucial for dynein-mediated organelle positioning. Post-translational modifications on dynein intermediate chain have been shown to be crucial for the formation of dynein-dynactin complexes.^39^^,^^48^^,^^49^ We found that DIC1, which is associated with dynactin subunit p150, is indeed ubiquitinated (Figure S7C), suggesting that ubiquitinated DIC1 forms active dynein-dynactin complexes. Moreover, to mechanistically understand how ARIH2 is linked with dynein mediated transport in cells, we hypothesized that ARIH2 mediated ubiquitination could regulate the interaction of dynein-dynactin. While DIC1 interaction with dynein heavy chain remains unchanged (Figure S7D), its interaction with p150 (dynactin subunit) gets significantly reduced upon ARIH2 depletion (Figures 8F and 8G), which explains the defective organelle positioning under similar conditions. This suggests that ARIH2 mediated ubiquitination on DIC1 regulates dynein function by facilitating the dynein-dynactin association in cells. In conclusion, our study identifies ARIH2 as an E3 ubiquitin ligase for dynein, revealing that ARIH2-mediated ubiquitination is essential for dynein activation. Furthermore, we demonstrate that the LisH protein Lis1 regulates ARIH2’s E3 ligase activity through the COP9 signalosome (CSN) complex, uncovering a previously unrecognized layer of control in dynein regulation (model in Figure 8H).

## Discussion

In this study, we present a comprehensive human LisH-domain-containing protein interaction network, constructed through systematic tandem affinity purification coupled with mass spectrometry (AP-MS). Our high-confidence dataset reveals a substantial proportion—approximately 90%—of previously uncharacterized interactions, alongside 137 known LisH-protein interactions, significantly expanding the current understanding of LisH-domain biology. Functional enrichment analysis of the interactome highlights previously unrecognized roles for LisH-domain-containing proteins in diverse cellular processes, suggesting that the functional landscape of this protein family is broader than previously appreciated.

LisH-domain-containing proteins have been implicated in fundamental biological processes such as microtubule dynamics, cell migration, nucleokinesis, and chromosome segregation,^50^ and thereby also contribute to the pathogenesis of different human diseases.^2^^,^^51^^,^^52^

While individual LisH proteins have been studied in isolation, there has been a lack of systematic efforts to map their collective interaction networks or to explore their integrative cellular functions. Our proteomic approach addresses this gap by offering an unbiased platform to dissect LisH protein connectivity on a global scale. Notably, we adopted a tandem affinity purification strategy to enhance specificity and reduce nonspecific background—a known limitation of traditional AP-MS workflows. This approach, successfully implemented in previous studies for identifying functional protein complexes^53^^,^^54^^,^^55^ has now facilitated the discovery of numerous previously unknown LisH-domain-containing protein interactions, hence reinforcing its reliability.

Furthermore, our work provided mechanistic insights into dynein regulation by Lis1. We uncover that the neddylation of the RBR E3 ligase ARIH2 enhances its capacity to ubiquitinate the dynein subunit DIC1—a mechanism that adds an additional layer to Lis1-mediated dynein regulation. While neddylation has been predominantly associated with the activation of Cullin-RING ligases (CRLs)^56^^,^^57^^,^^58^ and, to a lesser extent, with the HECT E3 ligase SMURF1,^59^ there have been no prior reports of RBR E3 ligases acting as direct substrates of neddylation. Although ARIH2 has been shown to be associated with neddylated Cullins for its activation, it has never been investigated as a potential neddylation substrate. This is the first report where neddylation is shown on an RBR E3 ligase, ARIH2. Just like Cullin neddylation activates CRLs to ubiquitinate their substrates, neddylation of ARIH2 also activates it and potentiates it to ubiquitinate dynein. Previously, the activation of ARIH2 has been shown in association with neddylated Cullin5 which relieves ARIH2 from its autoinhibition.^40^^,^^42^^,^^60^ However, our study reveals that neddylation on ARIH2 is sufficient for its activation, thereby showing an alternative strategy to avoid hampering the cellular ATP levels, which may be intensive in case of neddylated Cul5 route.

The functional consequences of the ARIH2-mediated ubiquitination of dynein are interesting. Although TRIM58 has been shown to ubiquitinate DIC2 and cause proteasomal degradation,^61^ but the precise mechanism and the functional significance of this modification seem inadequate. On the other hand, ubiquitination on dynein subunit DIC1 is unknown so far. Our study demonstrates ARIH2 as a DIC1 E3 ligase. Rather than promoting degradation, this ubiquitination appears essential for dynein activation and the maintenance of intracellular organelle positioning. This points to a non-proteolytic role for ubiquitination in regulating dynein function, opening up additional conceptual avenues for understanding motor protein regulation. Despite these advances, several important questions remain. The specific ubiquitination sites on DIC1, the structural and temporal details of ARIH2 activation by neddylation, and how these events coordinate with other modifications during Lis1’s dependent dynein regulation are not yet defined. Addressing these gaps in future studies will be crucial for developing a complete mechanistic model of dynein regulation.

Together, our findings offer a global framework for understanding LisH-domain protein interactions and introduce an additional mechanism of dynein regulation via neddylated ARIH2. These findings not only enhance the molecular understanding of Lis1-dynein biology but also suggest broader roles for neddylation and ubiquitination in intracellular transport and organelle dynamics. Future investigations building on this work will be critical for decoding the full spectrum of LisH protein functions and their implications in human health and disease.

### Limitations of the study

Our study demonstrates that the LisH protein interaction network uncovers several interactors and previously uncharacterized functions. While this proteomic screen identified many interacting partners, it does not provide quantitative information on the strength of these interactions. Further, the interactors identified here may vary across different cell types. We demonstrate that the neddylation of ARIH2 activates its ubiquitin ligase activity; however, the structural changes that relieves its autoinhibition remain unresolved. In addition, the precise sites and the type of ARIH2-mediated polyubiquitin linkage on DIC1 are yet to be defined. Finally, although we establish that DIC1 ubiquitination is essential for its association with dynactin, the precise mechanism underlying this process represents an important avenue for future investigation.

## Resource availability

### Lead contact

Requests for further information and resources should be directed to and will be fulfilled by the lead contact, Subbareddy Maddika (msreddy@cdfd.org.in).

### Materials availability

All unique/stable reagents generated in this study are available from the lead contact with a completed material transfer agreement.

### Data and code availability

• The mass spectrometry proteomics data have been deposited to the ProteomeXchange Consortium via the PRIDE^62^ partner repository with the dataset identifier PRIDE: PXD064011 https://doi.org/10.6019/PXD064011.
• No custom code was generated or used in this study.
• Any additional information required to reanalyze the data reported in this article is available from the lead contact upon request.

## Acknowledgments

This work was supported by 10.13039/501100001407DBT grant (BT/PR44301/BRB/10/2003/2021 to S.M), DST-ANRF grant (CRG/2022/008387 to S.M) and 10.13039/501100014128CDFD core funds. D.G. acknowledges the support of research fellowship [09/724(0129)/2017-EMR-I] from the 10.13039/501100001412Council of Scientific and Industrial Research (CSIR), India. We thank all members of LCDCS for their critical inputs. The authors thank Nanci Rani for providing technical assistance.

## Author contributions

S.M. conceptualized and managed the project. S.M. and D.G. designed the experiments, analyzed the data, and wrote the article. D.G. performed all the experiments.

## Declaration of interests

The authors declare no competing interests.

## STAR★Methods

### Key resources table

**Table:** 

REAGENT or RESOURCE	SOURCE	IDENTIFIER
**Antibodies**		

Rabbit Anti-Nedd8 (19E3)	Cell Signaling Technologies	Cat#2754 ; RRID:AB_659972
Rabbit Anti-Lis1	Novus Biologicals	Cat#NBP1-87769 ; RRID:AB_11059518
Mouse Anti-Lis1	Santa Cruz Biotechnology	Cat#sc-374586 ; RRID:AB_11008596
Mouse Anti-CSN1	Santa Cruz Biotechnology	Cat#sc-514086
Mouse Anti-CSN2	Santa Cruz Biotechnology	Cat#sc-136511 ; RRID:AB_10649207
Mouse Anti-CSN3	Santa Cruz Biotechnology	Cat#sc-100693 ; RRID:AB_2081616
Mouse Anti-CSN5	Santa Cruz Biotechnology	Cat#sc-13157 ; RRID:AB_627835
Mouse Anti-CSN7A	Santa Cruz Biotechnology	Cat#sc-398882
Mouse Anti-DHC	Santa Cruz Biotechnology	Cat#sc-514579
Rabbit Anti-DIC	Proteintech	Cat#12219-1-AP ; RRID:AB_2093630
Rabbit Anti-DYNC1LI2	Proteintech	Cat#18885-1-AP; RRID:AB_10596910
Mouse Anti-LAMP2	DSHB	Cat#GL2A7 ; RRID:AB_2314734
Mouse Anti-Flag	Sigma	Cat#F3165 ; RRID:AB_259529
Rabbit Anti-Flag	Sigma	Cat#F7425 ; RRID:AB_439687
Mouse Anti-β-Actin	Sigma	Cat#A5441 ; RRID:AB_476744
Mouse Anti-MBP	Sigma	Cat#M1321 ; RRID:AB_1079301
Mouse Anti- c-Myc (9E10)	Santa Cruz Biotechnology	Cat#sc40 ; RRID:AB_627268
Mouse Anti-EEA1	BD Biosciences	Cat#610456 ; RRID:AB_397829
Mouse Anti-GM130	BD Biosciences	Cat#610822 ; RRID:AB_398141
Mouse Anti-p230	BD Biosciences	Cat#611281 ; RRID:AB_398809
Goat Anti-p150	Abcam (kind gift from Dr Rashna Bhandari, CDFD)	Cat#Ab11806 ; RRID:AB_298590
Mouse Anti-ARIH1	Santa Cruz Biotechnology	Cat#sc-514551
Mouse Anti-ARIH2	Santa Cruz Biotechnology	Cat#sc-390682
Mouse Anti-Ubiquitin (P4D1)	Merck Millipore	Cat# 05-944 ; RRID:AB_441944
Mouse Anti-Ubiquitin (P4D1)	Santa Cruz Biotechnology	Cat#sc-8017 ; RRID: AB_628423
HRP Rabbit Anti-Mouse	Jackson Immunologicals	Cat#315-035-048 ; RRID:AB_2340069
HRP Goat Anti-Rabbit	Jackson Immunologicals	Cat#111-035-144
HRP Donkey Anti-Goat	Jackson Immunologicals	Cat#705-035-147 ; RRID:AB_2313587
FITC Goat Anti-Rabbit	Jackson Immunologicals	Cat#111-095-003 ; RRID:AB_2337972
Rhodamine Goat Anti-Mouse	Jackson Immunologicals	Cat#115-295-146 ; RRID:AB_2338766

**Chemicals, peptides, and recombinant proteins**		

MLN4924 (Pevonedistat)	Sigma	Cat#505477
1, 10 o-Phenanthroline	Sigma	Cat#131377
CSN5i3	MedChemExpress	Cat#HY-112134
COP9 Signalosome (human, purified)	Enzo Biochem	Cat#BML-PW9425-0020
Nedd8 E1 protein	R&D systems	Cat#E-313-025
Nedd8 UBE2M	R&D systems	Cat#E2-656-100
Nedd8 purified	R&D systems	Cat#UL-812-500

**Deposited data**		

Human LisH domain containing protein interaction network	ProteomeXchange Consortium via PRIDE^62^	PRIDE: PXD064011

**Experimental models: Cell lines**		

HEK 293T	ATCC	N/A
BOSC23	ATCC	N/A
U2OS	ATCC	N/A

**Oligonucleotides**		

Lis1 shRNA target sites 5’GCAGATTATCTTCGTTCAAAT3’ 5’TGACCATTAAACTATGGGATT3’	Horizon Discovery	TRCN0000050963 TRCN0000050966
ARIH2 shRNA target sites 5’CGACTCTGAAACAGCCAACTA3’ 5’GCTGGATGTGTCTAGGAGATT3’	Sigma	TRCN0000034271 TRCN0000034272

**Recombinant DNA**		

pENTR223 PAFAH1B1	DNASU	HsCD00515632
pLX304 FOPNL	DNASU	HsCD00439064
pDNR-Dual MAEA	DNASU	HsCD00003762
pET15_NSG TBL1X	DNASU	HsCD000583900
pLX304 TBL1XR1	DNASU	HsCD00446656
pVP16 ARMC9	DNASU	HsCD00083712
pLX304 TBL1Y	DNASU	HsCD00446642
pET15NESG RANBP9	DNASU	HsCD00585435
pLX304 TCOF1	DNASU	HsCD434268
pENTR223 MKLN1	DNASU	HsCD00516233
pDONR221 RMND5B	DNASU	HsCD00295628
pDONR221 SSBP2	DNASU	HsCD00043703
pDONR221 FGFR1OP	DNASU	HsCD00045089
pDONR221 NOLC1	DNASU	HsCD00043935
pDONR221 RMND5A	DNASU	HsCD00295665
pDONR 221 SSBP4	DNASU	HsCD00313439
pDONR 221 KIAA1468	DNASU	HsCD00813367
pDONR223 KATNAL2	DNASU	HsCD00353367
pDONR223 GID8	DNASU	HsCD00352788
pDONR223 SSBP3	DNASU	HsCD00352398
WDR47 cDNA	Dharmacon	4823902
pCR4-TOPO NPAT	Dharmacon	9020257/BC13620
pOTB7 WDR26	Dharmacon	6420398/BC052301
pOTB7 TAF5	Dharmacon	6575596/BC052268
pBluescriptR RANBP10	Dharmacon	30528481/BC099917
CSN1, CSN3, CSN4, CSN5, CSN6, CSN7A	Kind gift from Dr Lionel Pintard, Institute of Jacques Monod	Olma et al.^63^
CSN2 and CSN8	Kind gift from Dr Nicolas Thoma, Friedrich Miescher Institute of Biomedical Research	Lingaraju et al.^64^
DIC1 and DIC2	Kind gift from Dr Stuart Pitson, University of South Australia	Neubauer et al.^65^
eGFP-p150^glued^	Kind gift from Dr Santosh Chauhan, CCMB	–
ARIH1	This study	–
ARIH2	This study	–

**Software and algorithms**		

Software: GraphPad Prism8	GraphPad	Graphpad.com RRID: SCR_002798
Software: Adobe Photoshop 2021	Adobe	Adobe Creative Studio RRID:SCR_014199
Software: Adobe Illustrator 2023	Adobe	Adobe Creative Studio RRID:SCR_010279
Software: FIJI/ImageJ 1.54f	ImageJ	https://imagej.nih.gov/ij RRID: SCR_002285
Software: Cytoscape 3.10.2	Cytoscape	https://cytoscape.org/ RRID:SCR_003032
CRAPome	The Resource for Evaluation of Protein Interaction Networks (REPRINT)	https://reprint-apms.org/?q=chooseworkflow RRID:SCR_025008
ShinyGO	South Dakota State University	http://bioinformatics.sdstate.edu/go/ RRID:SCR_019213
ConsensusPathDB	Max Planck Institute for Molecular Genetics	http://cpdb.molgen.mpg.de/ RRID:SCR_002231
COMPLEAT	Harvard Medical School	Vinayagam et al.^23^

**Other**		

HMS Taplin Mass Spectrometry Core Facility	Harvard Medical School	https://taplin.hms.harvard.edu/ RRID:SCR_009813
Gel Doc Imaging system	BioRad	N/A
Leica TCS SP8	Leica	N/A
Chemidoc system	UV Tech, Cambridge	N/A

### Experimental model and study participant details

#### Cell lines

HEK293T, BOSC23, and U2OS cell lines were used in this study. These cell lines were grown in RPMI media containing 10% FBS and 1% penicillin and streptomycin under standard conditions at 37°C and 5% CO_2_. All cell lines were purchased from American Type Culture Collection (ATCC), which were tested and authenticated by the cell bank using their standard short tandem repeat (STR)-based techniques. Cell lines were routinely tested for mycoplasma contamination.

### Method details

#### Plasmids

The plasmids used in this study were cloned using Gateway Cloning (Invitrogen). Expression plasmids were moved into (S-protein/Flag/Streptavidin-binding-protein) SFB, Myc, GST, MBP and His tagged vectors for expression. All clones were verified by Sanger sequencing. All the point mutations were generated by PCR-based site directed mutagenesis and further cloned into Gateway vectors.

#### Transfection and transduction

Cell lines were transfected with various plasmids using PEI (Polyethylenimine) (Polysciences) according to the manufacturer’s protocol. Briefly, the plasmid was diluted in serum-free medium mixed with PEI (1 μg/μl) in 1:3 ratio. After incubating the DNA and PEI mixture at room temperature (RT) for 20 min, the complexes were added to cells to allow the transfection of plasmid. Lentivirus-based respective shRNA coding plasmids were transfected transiently using PEI (Invitrogen) in BOSC23 packaging cells along with packaging vectors (psPAX2 and pMD2.G). At 48 hrs post transfection, the viral media was collected, filtered through a 0.45 μM filter and added to the target cells along with polybrene (8 μg/ml). 48 hrs post transduction, cells were collected and processed for various assays and immunoblotting was performed with the specific antibodies to check the efficiency of knockdown. Knockdown stables were generated by selecting the cells with puromycin (2 μg/ml) 48 hours post transduction.

#### Tandem affinity purification

LisH domain containing proteins were purified by using tandem affinity purification as described previously.^53^ Briefly, HEK 293T cells expressing SFB-LisH domain containing proteins were lysed with NETN lysis buffer containing protease inhibitors on ice for 20 min. The cell lysates were incubated with streptavidin–sepharose beads for 2 h at 4 °C on end-to-end rotation. Then, beads were washed with NETN lysis buffer and the associated proteins were eluted using 2 mg·mL^−1^ Biotin (Sigma) for 2 h at 4 °C on end-to-end rotation. The eluates from the first step of purification were then incubated with S-protein–agarose beads (Novagen) for 2 h at 4 °C on end-to-end rotation. After clearing the unbound proteins by washing, the proteins were eluted by boiling in SDS-loading buffer for 5 min at 95 °C. Eluted protein lysate was loaded on SDS-PAGE. The associated proteins were identified by in-gel trypsin digestion followed by LC-MS/MS analysis at Taplin Biological Mass Spectrometry Facility (Harvard University). SFB vector and SFB-tagged GFP were used as controls for purification.

#### Protein sequence analysis by LC-MS/MS

This method has been described previously.^53^ Briefly, after excising the gel bands they were subjected to a modified in-gel trypsin digestion procedure. Gel pieces were washed and dehydrated with acetonitrile for 10 min followed by removal of acetonitrile. Pieces were then completely dried in a speed-vac. Rehydration of the gel pieces was carried out with 50 mM ammonium bicarbonate solution containing 12.5 ng/μl modified sequencing-grade trypsin (Promega, Madison, WI) at 4 °C. After 45 min, the excess trypsin solution was removed and replaced with 50 mM ammonium bicarbonate solution to just cover the gel pieces. Samples were then placed in a 37 °C room overnight. Peptides were later extracted by removing the ammonium bicarbonate solution, followed by one wash with a solution containing 50% acetonitrile and 1% formic acid. The extracts were then dried in a speed-vac (∼1 h). The samples were then stored at 4°C until analysis. On the day of analysis, the samples were reconstituted in 5−10 μL of HPLC solvent A (2.5% acetonitrile, 0.1% formic acid). A nanoscale reverse-phase HPLC capillary column was created by packing 2.6 μm C18 spherical silica beads into a fused silica capillary (100 μm inner diameter ∼ 30 cm length) with a flame-drawn tip. After equilibrating the column, each sample was loaded via a Famos auto sampler (LC Packings, San Francisco CA) onto the column. A gradient was formed, and peptides were eluted with increasing concentrations (gradient of 2% to 30%) of solvent B (97.5% acetonitrile, 0.1% formic acid) for 1 h. As peptides eluted, they were subjected to electrospray ionization and then entered into an LTQ Orbitrap Velos Pro ion-trap mass spectrometer (Thermo Fisher Scientific, Waltham, MA). Peptides were detected, isolated, and fragmented to produce a tandem mass spectrum of specific fragment ions for each peptide. Peptide sequences (and hence protein identity) were determined by matching protein databases from UniProt with the acquired fragmentation pattern by the software program, Sequest (Thermo Fisher Scientific, Waltham, MA). All databases include a reversed version of all the sequences, and the data were filtered to one percent peptide false discovery rate.

#### Data filtering and data analysis

The interactome data was filtered using CRAPome tools and as described before.^53^ SAINT score more than 0.7 and FCA more than 1.8, FCB > 2, and IS > 1 was used to filter data. Bait and prey associations retained after SAINT score cutoff were further subjected to mapping using UniProt ID mapping and HGNC gene symbols in order to represent them using most recent protein identifiers. Uniform protein identifier representation was used across different sets of PPIs identified in this study as well as those retrieved from iRefIndex and IntAct. From this only human PPIs were considered (both interactors are human proteins). Normalized Spectral Abundance Factor (NSAF) was calculated by dividing the number of spectral counts (SpC) identifying a protein by its length (L) in amino acids, and then normalizing this value by the sum of SpC/L for all proteins identified in the experiment.

NSAF = (Spectral Counts / Protein Length) / Sum of (Spectral Counts / Protein Length) for all proteins.

KEGG pathway enrichment was done using ConsensusPathDB, and pathway with p value < 0.001 was selected. GO analysis was done using ShinyGO, and protein complexes were identified by using COMPLEAT. Network was visualized using Cytoscape. Heat maps were made using Morpheus.

#### Affinity-based pulldown and immunoprecipitation

Cells were lysed with NETN buffer (20 mM Tris/HCl, pH 8.0, 100 mM NaCl, 1 mM EDTA, and 0.5% Nonidet P-40) containing 1 mg·mL^−1^ of each Pepstatin A, Aprotinin, and 100 mM PMSF (Phenyl Methyl Sulfonyl Fluoride) on ice for 20 minutes. The whole-cell lysates were incubated with either Protein G Sepharose beads conjugated with respective antibody (2 ug), or Streptavidin–Sepharose beads (Amersham Biosciences), or S-protein–agarose beads (Novagen) for 2 h at 4°C. The protein complexes were then washed with NETN buffer and subjected to SDS-PAGE. Immunoblotting was performed using standard protocols.

For detecting neddylation and ubiquitination in cells, denaturing immunoprecipitation was performed by boiling the HEK 293T cells in denaturing lysis buffer (50 mM Tris-HCl pH 7.5, 100 mM β-mercaptoethanol, 1% SDS and 5 mM EDTA) for 10 min followed by sonication. The SDS concentration was adjusted to 0.1% by adding 1× NETN buffer and incubated on ice for 20 min. The whole-cell lysates obtained by centrifugation were incubated with Streptavidin–Sepharose for overnight at 4°C. The immunocomplexes were then washed with NETN buffer and subjected to SDS–PAGE.

For detecting neddylation, 2 μM of 1,10 OPT (o-phenanthroline) was added to the lysis buffer. For *in vivo* ubiquitination, the cell lysate was denatured and the substrate protein was pulled down using Streptavidin-Sepharose beads (as described above). The substrate protein was eluted from the beads by boiling in 2X SDS dye. The samples were loaded on 7.5% Tris-Glycine gels and were resolved until the size of the substrate protein. The gels were then transferred on PVDF membrane and probed with substrate-specific or substrate-tagged antibody. The ubiquitinated substrate can be visualized as a smear by chemiluminescence.

#### Recombinant protein purification and enzymatic assay

GST, MBP and His tagged proteins were transformed into *Escherichia coli* BL21 (DE3) cells. Cultures were grown to an optical density (OD) at 600 nm of ∼0.6 and induced with 0.5 mM isopropyl β-D-1-thiogalactopyranoside (IPTG) at 18°C for overnight. The cell pellets were lysed in lysis buffer (50 mM Tris-HCl pH 7.5, 150 mM NaCl, and 0.01% NP–40 IGEPAL), and 20 mM Imidazole pH 8.0 with 0.1% NP-40 IGEPAL (for His-tagged proteins), and protease inhibitors (Aprotinin, Pepstatin, PMSF) and sonicated. Cell lysates were pulled down with Dextran–Sepharose or Glutathione Sepharose or Ni-NTA beads for 2 h at 4°C. Then, beads were washed five times with wash buffer (50 mM Tris-HCl pH 7.5, 300 mM NaCl, 0.01% NP–40 IGEPAL, 1 mM DTT and protease inhibitors) or and bound proteins were eluted with the elution buffer containing 20 mM Tris-HCl pH 7.5, 200 mM NaCl, 1 mM EDTA and 10 mM maltose, or 50 mM Tris-HCl pH 8, 150 mM NaCl and 10 mM reduced glutathione, or 50 mM Tris-HCl pH 7.5, 150 mM NaCl, 300 mM Imidazole pH 8.0 with 0.1% NP-40 IGEPAL. The proteins after purification were used for further enzymatic assay.

For *in vitro* neddylation assay, 1 μM NAE1, 2 μM UBE2M, and 15 μM Nedd8 were added to reactions with neddylation buffer (50 mM Tris, pH 7.6, 150 mM NaCl, 10 mM MgCl_2_, 1 mM DTT and 5 mM ATP). Reactions were incubated at 30°C for 1 h, terminated with SDS loading buffer and analyzed by SDS-PAGE. For deneddylation reactions, the initial neddylation reaction was washed with neddylation buffer twice, and incubated for another 1 h at 30°C with purified CSN complex (0.3 μg), a modified protocol.^66^

#### Immunofluorescence

Cells grown on coverslips were fixed with 4% paraformaldehyde for 10 min at room temperature. After washing with PBS, the cells were permeabilized with 0.2% Triton X-100 for 10 min followed by blocking with 5% BSA at room temperature for 30 min and incubation with primary antibodies for overnight at 4°C. After incubation, cells were washed three times with PBS and then incubated with FITC or Rhodamine conjugated secondary antibodies at room temperature for 60 min followed by three washes with PBS. Cells were washed with PBS, and coverslips were mounted with glycerol containing paraphenylenediamine and imaged using a confocal microscope (Leica TCS SP8).

#### Image analysis and quantification

##### Organelle positioning (lysosomes/endosomes)

Quantification of the distribution of lysosomes and endosomes based on LAMP2 and EEA1 signal intensity was performed as described in.^67^ Briefly, a boundary was drawn around the nucleus and intensity were measured. This boundary was then incremented with concentric circles until the periphery of the cell. The intensity was measured for each concentric circle ROI. The perinuclear accumulation was calculated by subtracting the second ROI from the first, and the peripheral accumulation was calculated by subtracting the last ROI from the second last. Dividing these numbers by the total intensity of the respective cell gives the distribution of lysosomes and endosomes of each cell.

##### Organelle positioning (golgi apparatus)

The golgi morphology was described by observing visual changes in its distribution for each cell across the samples. The standard for each category of compact and scatter phenotypes has been shown in relevant studies.^46^

### Quantification and statistical analysis

All the graphs in this study represent either mean ± SD, or mean ± SE, or median ± interquartile range (mentioned in the respective figure legends, wherever applicable) and p values were calculated using two-tailed Student’s t-test, One-way ANOVA, or Two-way ANOVA (GraphPad Prism 8.0). Differences between groups were considered statistically significant for p values < 0.05. Proteomic data were analyzed from two biological replicates. All other biochemical and immunofluorescence data were analyzed from at least three independent experiments.

## Supplemental information

Document S1. Figures S1–S7

Table S1. LisH proteins from UniProt: Tab A) List of LisH-domain containing proteins identified from UniProt database. (Related to Figure 1)

Table S2. Systematic analysis of LisHome from CRAPome: Tab A) Raw, and Tab B) filtered (HCIP) list of interactors shown with the respective interaction scores. (Related to Figure 1)

Table S3. Correlation of NSAF of LisH protein with HCIs: Tab A) A table of normalized spectral abundance factor (NSAF) for HCIs of each LisH protein obtained from the network. Tab B) Calculations for determining NSAF are shown. (Related to Figure 1)

Table S4. Distribution of HCIs with respect to LisH proteins: Tab A) A matrix of fold change scores of baits with the HCIPs obtained from the network. (Related to Figure 1)

Table S5. Gene Ontology analysis of LisHome: Tab A) List of GO terms of biological process and Tab B) cellular component for the interactors obtained from the network. (Related to Figure 2)

Table S6. KEGG pathway analysis of LisHome: Tab A) List of KEGG pathways enriched from ConsensusPathDB for the interactors obtained from the network. (Related to Figure 2)

Table S7. Enrichment of multiprotein complexes from LisHome: Tab A) List of identified multiprotein complexes enriched for the interactors obtained from the network. (Related to Figure 3)

Table S8. Enrichment of proteins linked to genetic disorders from LisHome: Tab A) List of associated genes linked with genetic diseases obtained from OMIM within the LisH interactome network. (Related to Figures 3 and S3)

Table S9. Enrichment of cancer-linked genes from LisHome: Tab A) List of all the cancer-linked genes obtained from the COSMIC database within the LisH interactome network. Tab B) List of cancer linked genes specific to the LisH proteins are shown. (Related to Figure S3)
