# Supplementary material for: A LisH-domain protein interaction map reveals a Lis1-ARIH2-dynein regulatory axis
Source: iScience. 2025 Oct 30;28(11):113912. doi: 10.1016/j.isci.2025.113912 (PMC12648486; doi:10.1016/j.isci.2025.113912)
Supplement: Document S1. Figures S1–S7 [file mmc1.pdf]

iScience, Volume *28*

Supplemental information

A LisH-domain protein interaction map reveals a Lis1-ARIH2-dynein regulatory axis

Devanshi Gupta and Subbareddy Maddika

Supplementary Data Figures

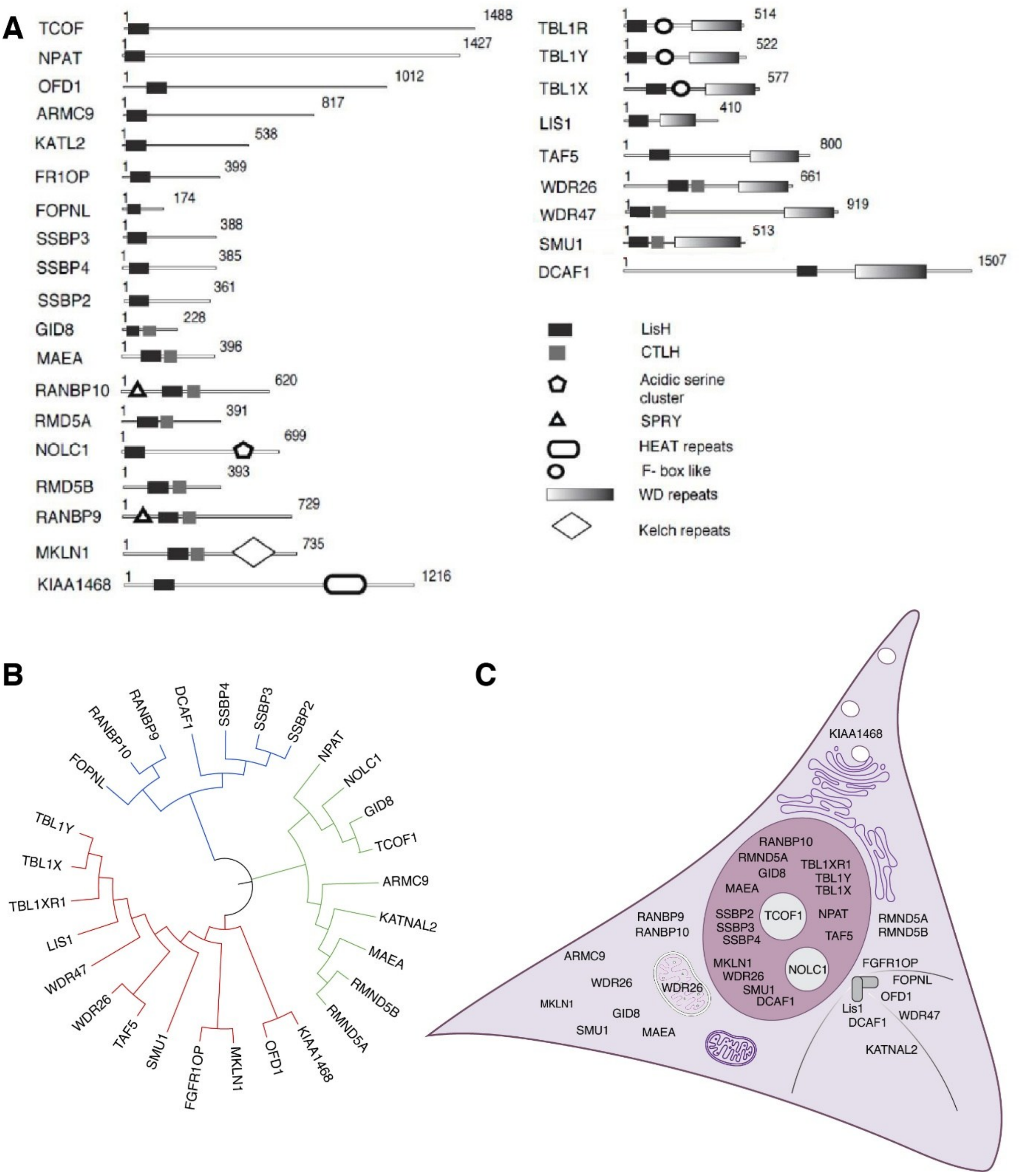

Supplementary Figure 1: LisH-domain containing proteins in humans. (A) Domain architecture of proteins containing LisH domain (ID: PS50896) identified through UniProt database in human. **(B)** Phylogenetic tree of LisH-domain containing proteins in humans. **(C)**

Pictorial representation of cellular localization of LisH-domain containing proteins in humans based on the UniProt database. **(Related to Figure 1)**

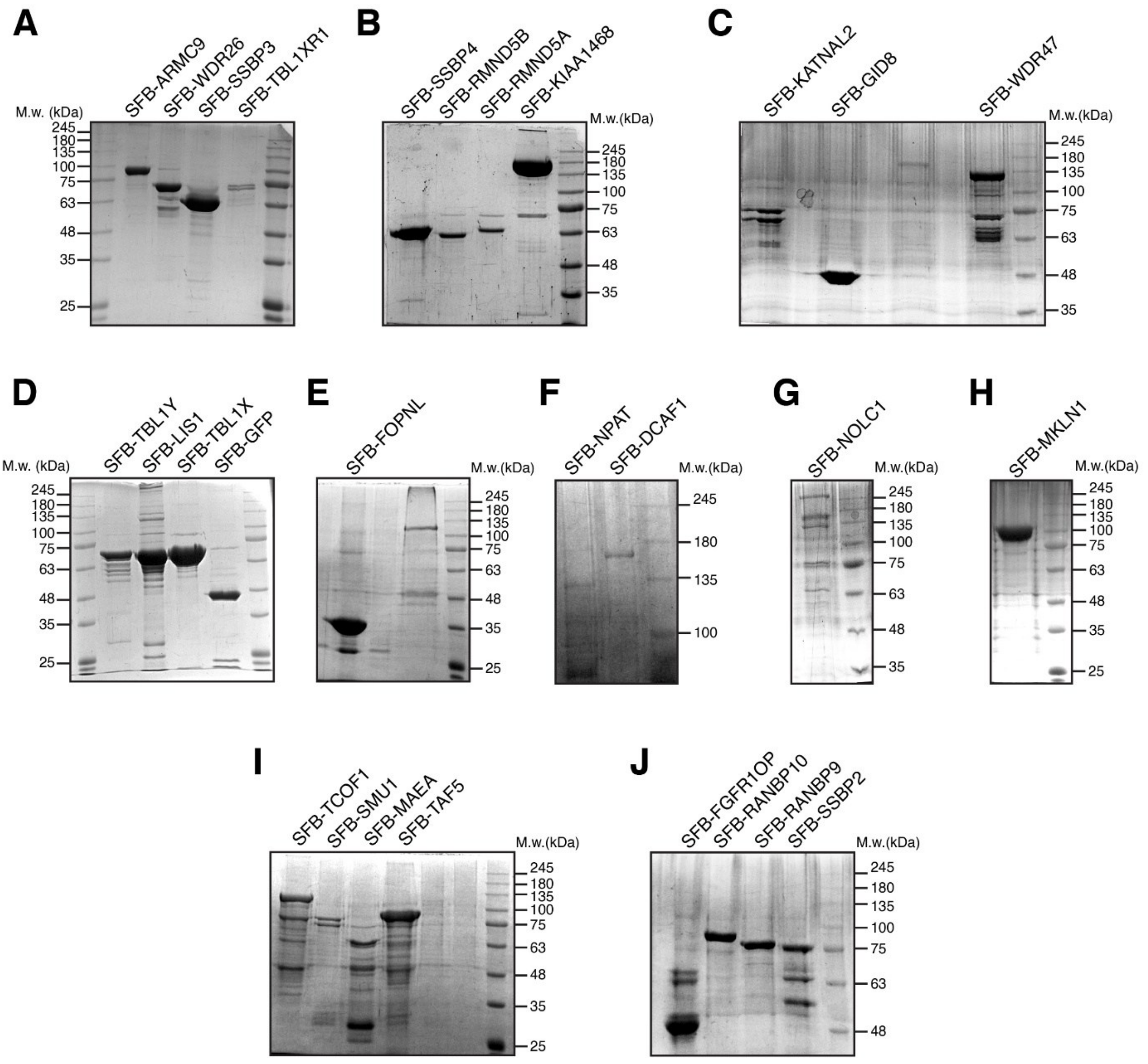

Supplementary Figure 2: Purified interactome of LisH-domain containing proteins. (A - J) Coomassie stained SDS-PAGE gels showing purified interactome of each LisH-domain containing proteins from HEK 293T cells. **(Related to Figure 1)**

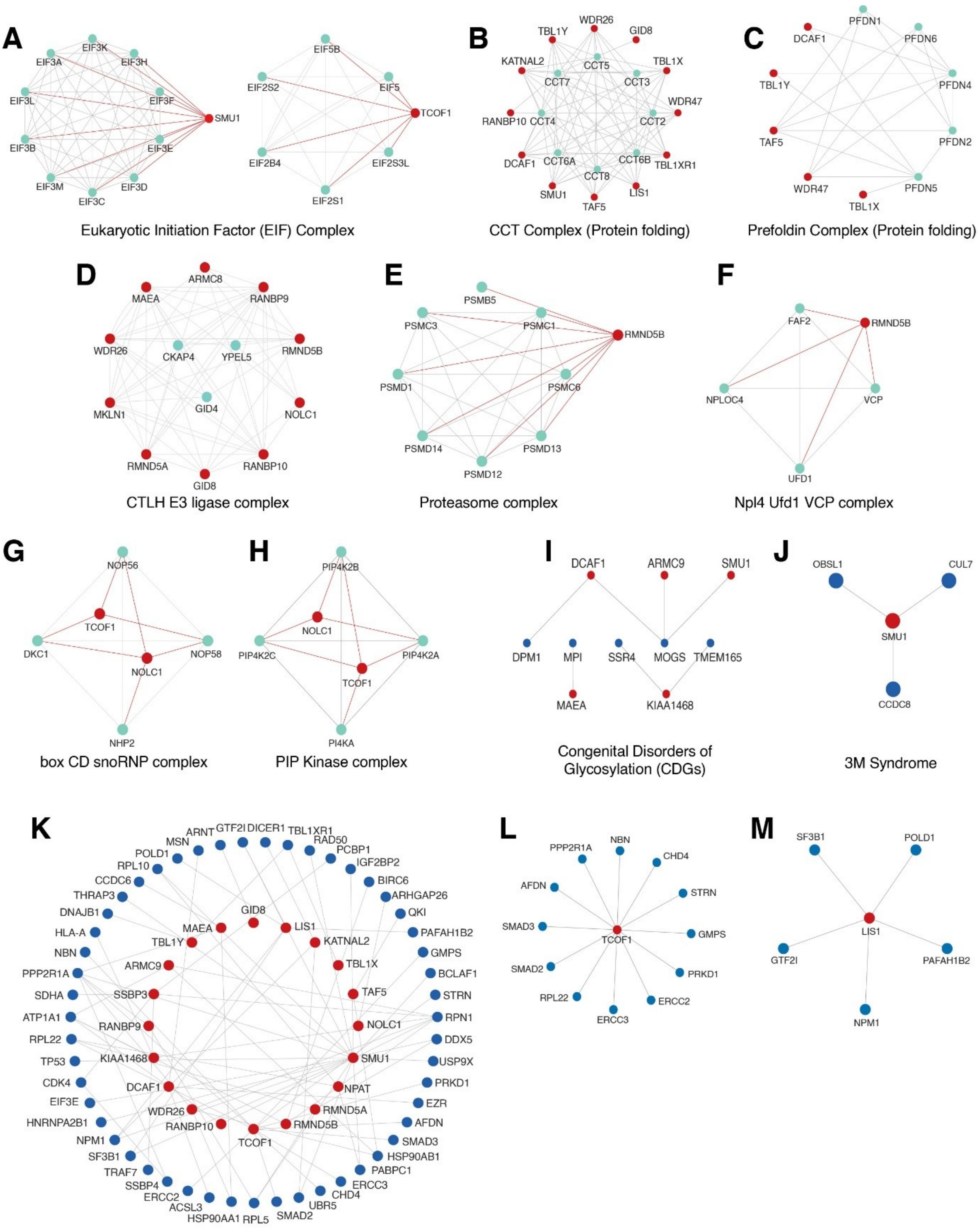

Supplementary Figure 3: Relationship between interactors from LisHome. The interactors from each LisH-domain containing protein were searched against the COMPLEAT database and the representative LisH protein−multiprotein complexes like **(A)** eukaryotic initiation factor complexes, **(B)** CCT complex, **(C)** Prefoldin complex, **(D)** CTLH E3 ligase complex,

(E) Proteasome complex, **(F)** Npl4-Ufd1-VCP complex, **(G)** box CD snoRNP complex, **(H)** PIP kinase complex, are shown. The circles in red are the LisH proteins, while circles in green are the interactors. Interaction network of LisH proteins with genetic diseases like **(I)** Congenital disorders of glycosylation (CDGs), and **(J)** 3M syndrome components are shown. **(K)** Interaction network of LisH proteins and cancer-linked proteins (from COSMIC database) built by cytoscape. **(L)** Cancer-linked proteins in complex with TCOF1 and **(M)** Lis1 are indicated. The circles in red are the LisH proteins, while circles in blue are the interactors. **(Related to Figure 2 & 3)**

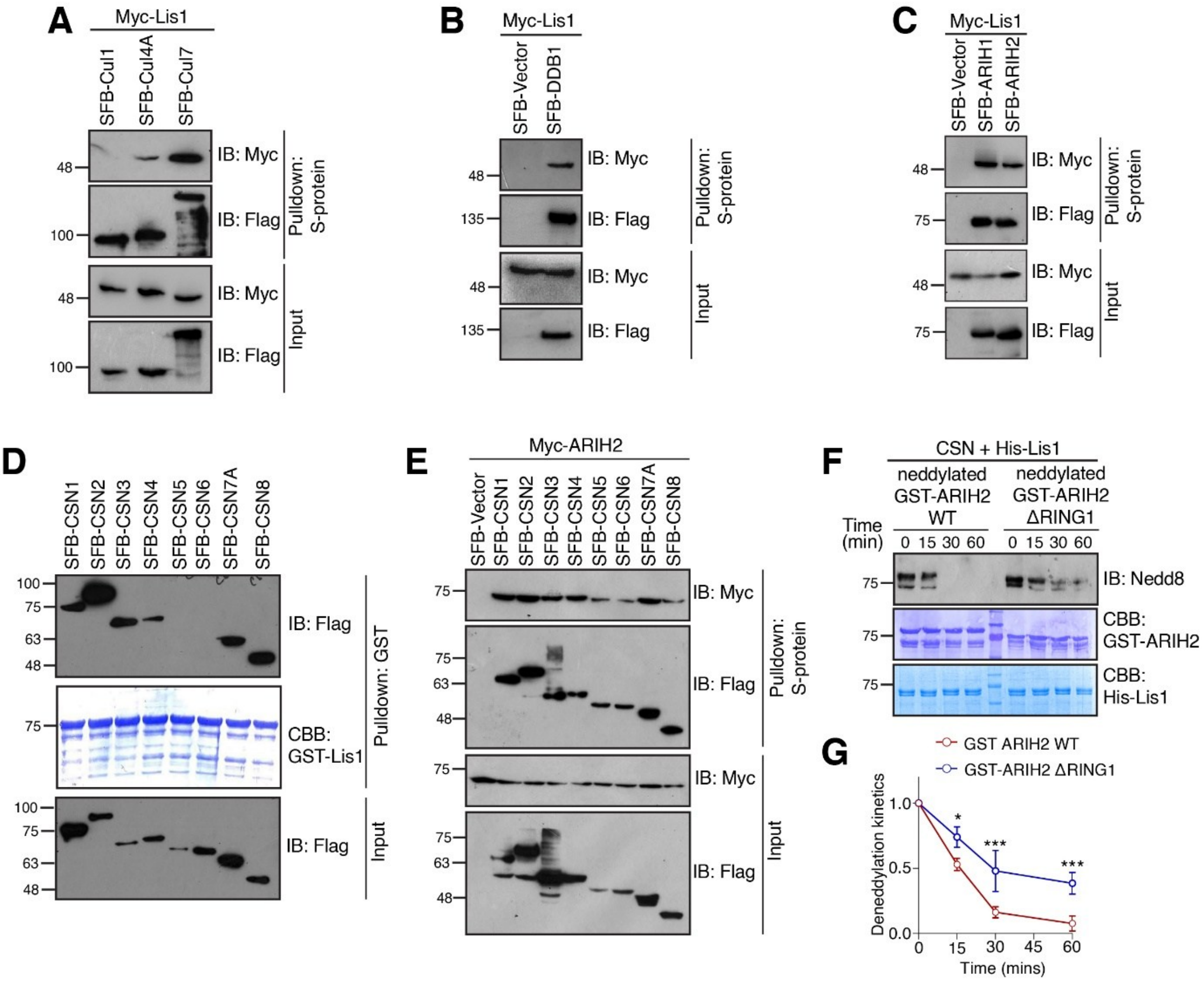

Supplementary Figure 4: Association of Lis1 with CSN and ARIH2. (A) HEK 293T cells were transfected with SFB Cul1, SFB Cul4A, and SFB-Cul7 along with Myc Lis1 plasmids. Cells were lysed, and incubated with S-protein beads. Interaction was detected by immunoblotting with myc antibody. **(B)** HEK 293T cells were transfected with SFB DDB1 along with Myc Lis1 plasmids. Cells were lysed, and incubated with S-protein beads. Interaction was detected by immunoblotting with myc antibody. **(C)** HEK 293T cells were transfected with SFB ARIH1 and SFB ARIH2 along with Myc Lis1 plasmids. Cells were lysed, and incubated with S-protein beads. Interaction was detected by immunoblotting with myc antibody. **(D)** HEK 293T cells were transfected with SFB CSN1, SFB CSN2, SFB CSN3, SFB CSN4, SFB CSN5, SFB CSN6, SFB CSN7A and SFB CSN8 plasmids. Cells lysates were incubated with purified GST Lis1 on beads for 2 hours. Interaction was detected by

immunoblotting with Flag antibody. **(E)** HEK 293T cells were transfected with SFB CSN1, SFB CSN2, SFB CSN3, SFB CSN4, SFB CSN5, SFB CSN6, SFB CSN7A and SFB CSN8 along with Myc ARIH2 plasmids. Cells were lysed, and incubated with S-protein beads. Interaction was detected by immunoblotting with myc antibody. **(F)** Bacterially purified GST-ARIH2 WT and ΔRING1 was incubated with NAE1, UBC12 and Nedd8 for in vitro neddylation for 60 mins, and with purified CSN in combination with His-Lis1 for in vitro deneddylation for the indicated time durations. Neddylated protein was visualized by immunoblotting with anti-Nedd8 antibody. **(G)** Quantification for the experiment shown in (F). Data are represented as mean +/- SEM from three biological replicates (*p = 0.0139, ***p = 0.0004, ***p = 0.0005; Two-way ANOVA followed by Dunnett's multiple comparisons test). **(Related to Figure 4 & 5)**

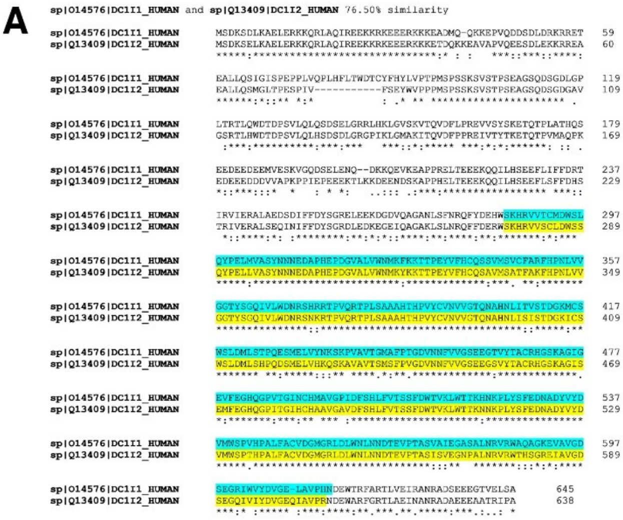

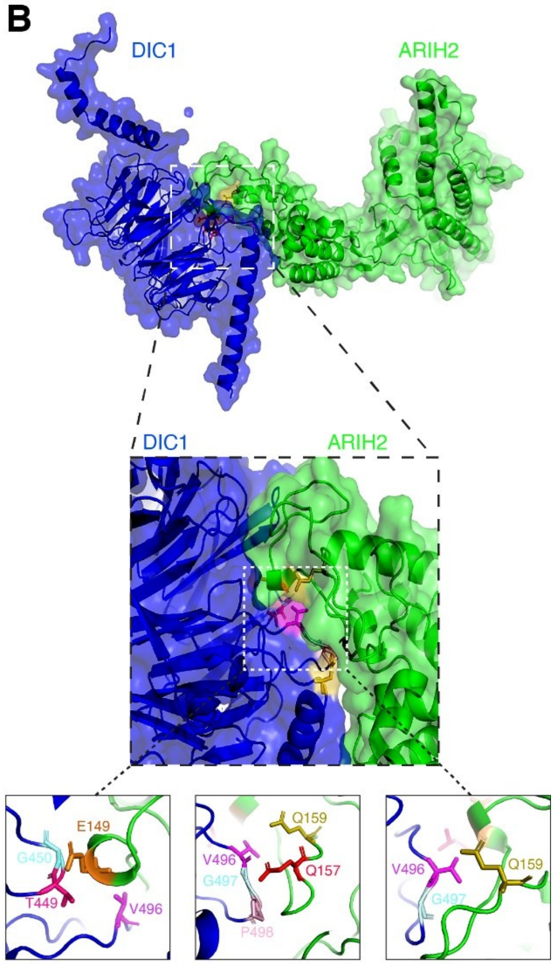

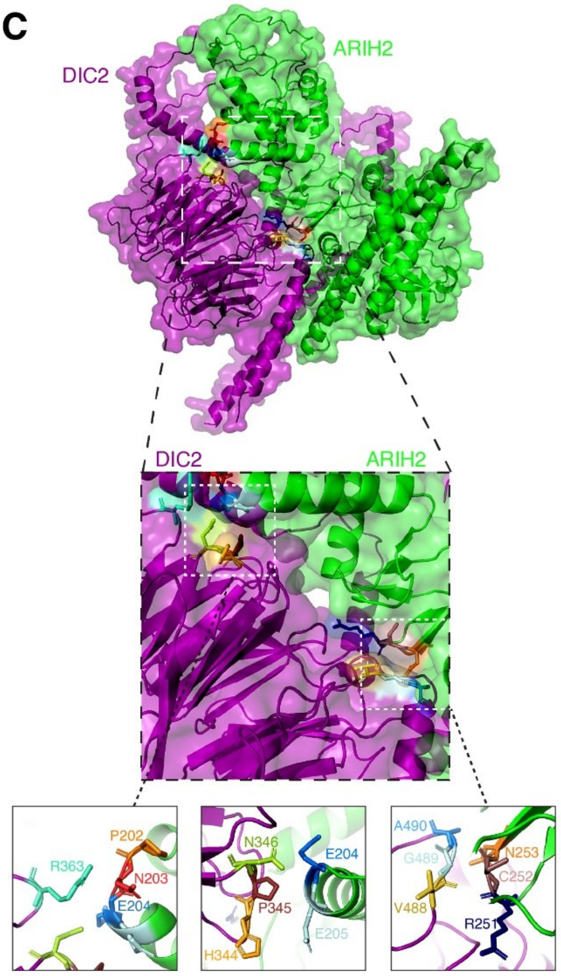

D

	Docking Score	Confidence Score
DIC1 - ARIH2	-275.01	0.9242
DIC2 - ARIH2	-235.07	0.8457

Supplementary Figure 5: Characterization of binding of DIC1 and DIC2 with ARIH2. **(A)** A multiple sequence alignment of Human DIC1 and DIC2 obtained by ClustalW is shown. **(B)** Molecular docked structures of DIC1 and ARIH2 obtained via Alphafold2 and HDOCK, indicating the contact sites of the binding interface. The blue colour represents DIC1, while

green represents ARIH2. **(C)** Molecular docked structures of DIC2 and ARIH2 obtained via Alphafold2 and HDOCK, indicating the contact sites of the binding interface. The pink colour represents DIC2, while green represents ARIH2. **(D)** Table describing the docking and confidence scores of molecular docked DIC1:ARIH2 and DIC2:ARIH2 structures obtained via HDOCK. **(Related to Figure 7)**

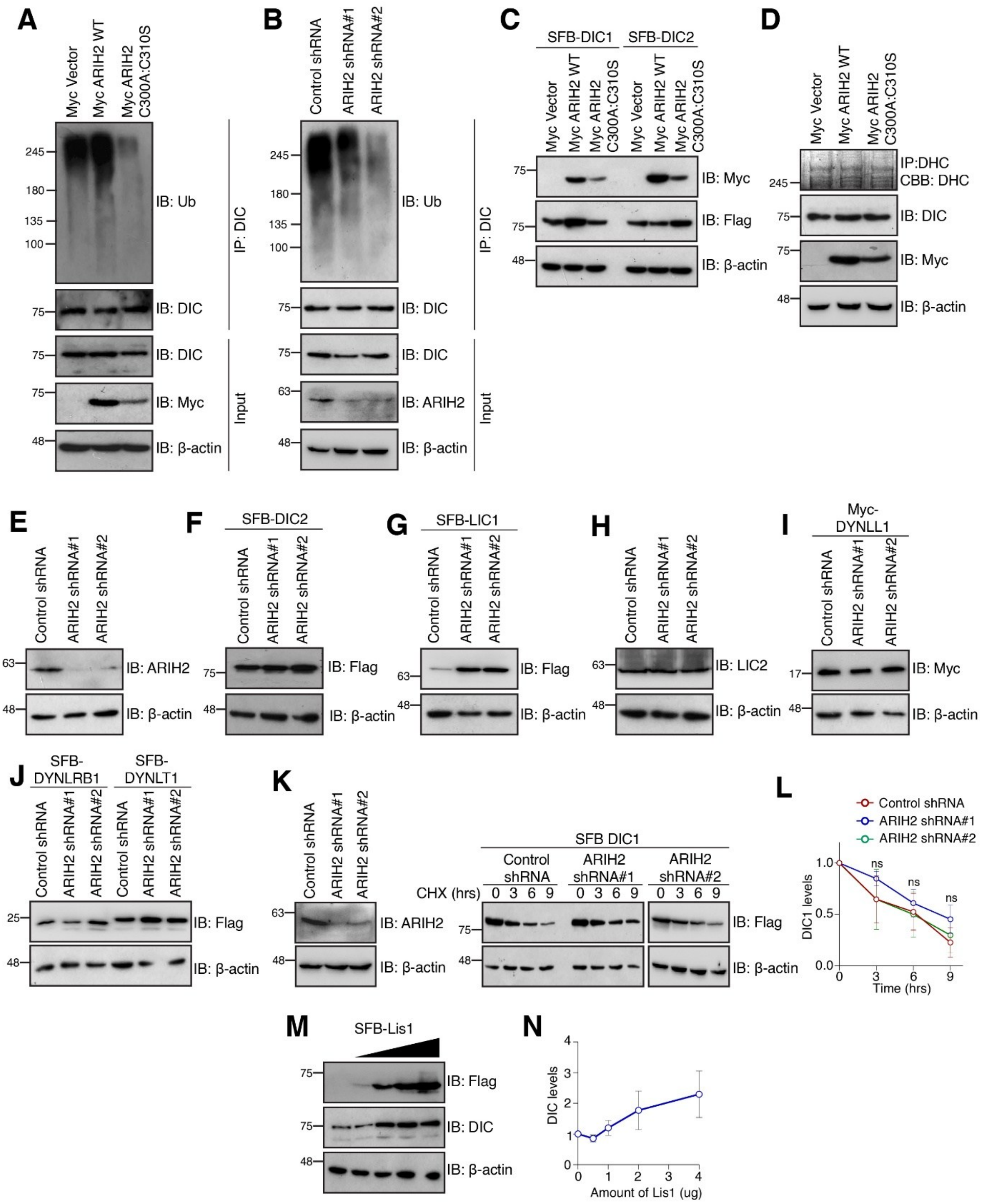

Supplementary Figure 6: ARIH2 mediated ubiquitination on DIC1 is non-proteolytic. (A) HEK293T cell were transfected with Myc-Vector, Myc-ARIH2 WT and Myc-ARIH2 C300A:C310S plasmids. Cell lysates were subjected to denaturing immunoprecipitation and incubated with DIC antibody. Presence of ubiquitination was detected by western blotting with respective antibodies. **(B)** Cell lysates from Control and ARIH2 depleted cells were subjected

to denaturing immunoprecipitation and incubated with DIC antibody. Presence of ubiquitination was detected by western blotting with respective antibodies. **(C)** HEK293T cell were transfected with Myc-Vector, Myc-ARIH2 WT and Myc-ARIH2 C300A:C310S, along with either SFB-DIC1 or SFB-DIC2 plasmids. Protein levels at steady state were detected by immunoblotting with respective antibodies**.** **(D)** HEK293T cell were transfected with Myc-Vector, Myc-ARIH2 WT and Myc-ARIH2 C300A:C310S plasmids. Protein levels at steady state were detected by immunoblotting with respective antibodies**.** DHC levels were visualized by immunoprecipitation using DHC antibody followed by staining with Coomassie Brilliant Blue (CBB). **(E)** ARIH2 was depleted in HEK 293T cells using ARIH2-specific shRNAs targeting at different sites and a stable line was generated. The knockdown was confirmed by probing with ARIH2 antibody. Control and ARIH2 depleted stable HEK 293T cells were transfected with **(F)** SFB-DIC2, **(G)** SFB-LIC1, **(H)** endogenous LIC2, **(I)** Myc-DYNLL1, **(J)** SFB-DYNLRB1 and SFB DYNLT1, individually. Protein levels at steady state were detected by immunoblotting with respective antibodies**.** **(K)** Control and ARIH2 depleted stable HEK293T cells were transfected with SFB-DIC1 plasmid. 24 hours after transfection, cycloheximide (CHX) was added at 50 ug/ml and chased until indicated time points. Protein levels were detected by immunoblotting with respective antibodies. **(L)** Quantification for the experiment done in (K) through three biological replicates is shown. Data are represented as mean +/- SEM (ns: not significant). **(M)** HEK293T cells were transfected with increasing concentrations (0, 0.5, 1, 2, 4 ug respectively) of SFB-Lis1 plasmid. Protein levels at steady state were detected by immunoblotting with respective antibodies**.** The quantification from three independent experiments is shown in **(N)**. Data are represented as mean +/- SEM. **(Related to Figure 7)**

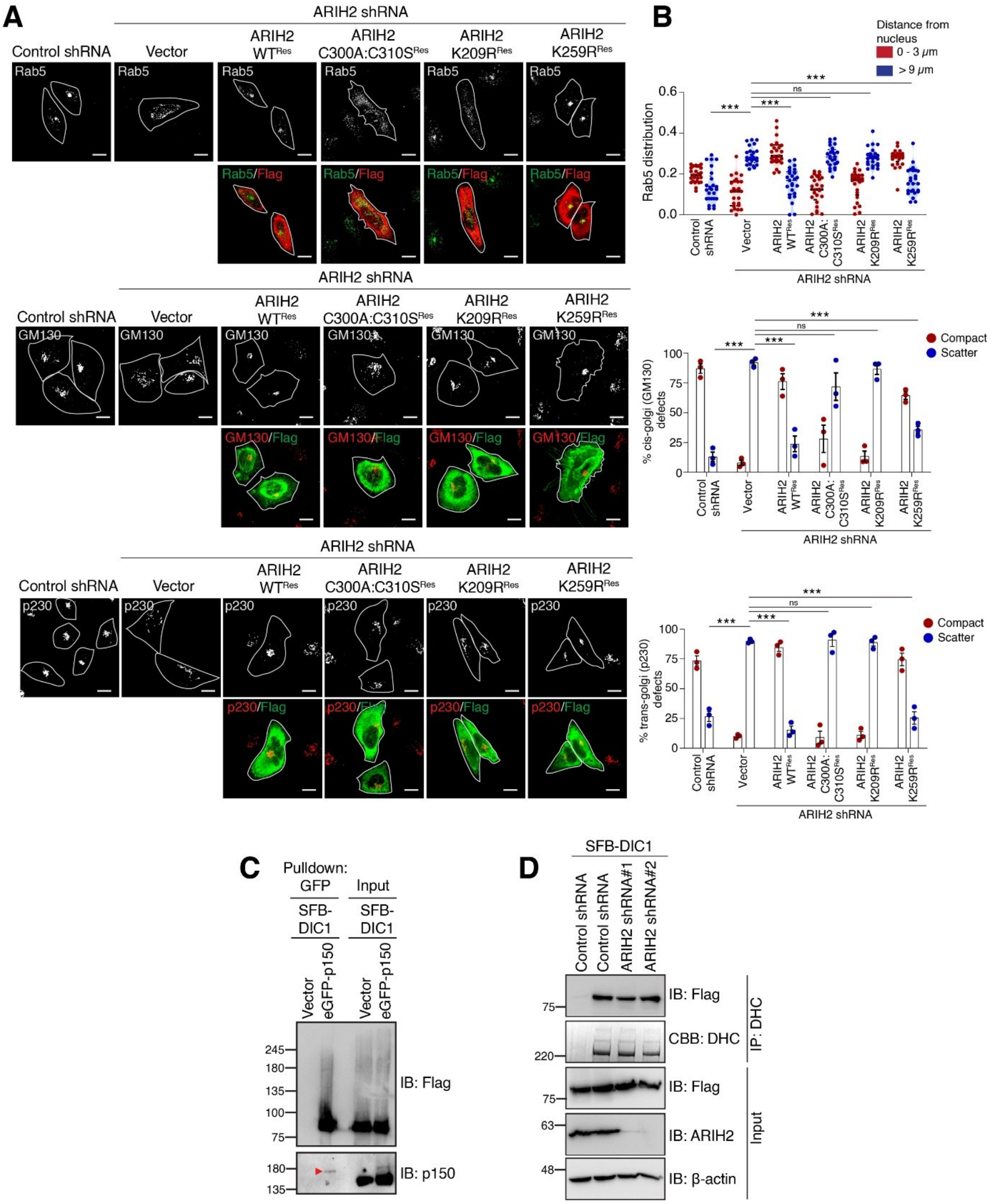

Supplementary Figure 7: ARIH2 mediated ubiquitination on DIC1 is essential for dynein function. (A) Control and ARIH2 depleted U2OS cells were transfected with shRNA resistant SFB ARIH2 WT, SFB ARIH2 C300A:C310S, SFB ARIH2 K209R, and SFB ARIH2 K259R plasmids. The cells were fixed and labelled with Rab5 (early endosome), GM130 (cis-Golgi) and p230 (trans-Golgi) antibodies to visualize their distribution under confocal microscope.

Representative images from three independent experiments are shown. Scale bars: 10 µm. **(B)** Quantification of the distribution of Rab5-positive compartments (n=25) in U2OS cells for the experiments shown in (A). Data represents median ± interquartile range from three independent experiments. (***p < 0.0001; ns = not significant; One way ANOVA followed by Dunnett's multiple comparisons test). The golgi distribution has been categorized into 2 types: compact and scatter. The values plotted are the mean ± SE from three independent experiments. (***p < 0.0001; ns = not significant; Two-way ANOVA followed by Dunnett's multiple comparisons test). **(C)** HEK 293T cells were transfected with SFB DIC1 along with Vector and eGFP-p150 plasmids. Cells were lysed, and incubated with GFP-agarose beads. Ub smear of SFB-DIC1 was detected by immunoblotting with Flag antibody. **(D)** Control and ARIH2 depleted HEK 293T cells were transfected with SFB DIC1 plasmid. Cells were lysed, and incubated with IgG or DHC-bound Protein G beads. Interaction was detected by immunoblotting with Flag antibody. DHC pulldown is visualized by Coomassie Brilliant Blue (CBB) staining. **(Related to Figure 8)**
